# Diverse *Colletotrichum* species cause anthracnose of tea plants (*Camellia sinensis* (L.) O. Kuntze) in China

**DOI:** 10.1038/srep35287

**Published:** 2016-10-26

**Authors:** Yu-Chun Wang, Xin-Yuan Hao, Lu Wang, Bin Xiao, Xin-Chao Wang, Ya-Jun Yang

**Affiliations:** 1 Tea Research Institute, Chinese Academy of Agricultural Sciences/National Center for Tea Improvement/Key Laboratory of Tea Biology and Resources Utilization, Ministry of Agriculture, Hangzhou 310008, People’s Republic of China; 2 College of Horticulture, Northwest A&F University, Yangling 712100, Shaanxi, People’s Republic of China

## Abstract

Anthracnose caused by *Colletotrichum* is one of the most severe diseases that can afflict *Camellia sinensis*. However, research on the diversity and geographical distribution of *Colletotrichum* in China remain limited. In this study, 106 *Colletotrichum* isolates were collected from diseased leaves of *Ca. sinensis* cultivated in the 15 main tea production provinces in China. Multi-locus phylogenetic analysis coupled with morphological identification showed that the collected isolates belonged to 11 species, including 6 known species (*C. camelliae*, *C. cliviae*, *C. fioriniae*, *C. fructicola*, *C. karstii*, and *C. siamense*), 3 new record species (*C. aenigma*, *C. endophytica*, and *C. truncatum*), 1 novel species (*C. wuxiense*), and 1 indistinguishable strain, herein described as *Colletotrichum* sp. Of these species, *C. camelliae* and *C. fructicola* were the dominant species causing anthracnose in *Ca. sinensis*. In addition, our study provided further evidence that phylogenetic analysis using a combination of ApMat and GS sequences can be used to effectively resolve the taxonomic relationships within the *C. gloeosporioides* species complex. Finally, pathogenicity tests suggested that *C. camelliae*, *C. aenigma*, and *C. endophytica* are more invasive than other species after the inoculation of the leaves of *Ca. sinensis*.

The tea plant (*Camellia sinensis* (L.) O. Kuntze) is an important non-alcoholic beverage crop that is widely cultivated in tropical and subtropical areas. China, one of the earliest countries to begin cultivating tea plants, cultivates the widest variety of tea plants over the largest area in the world, spanning 20 southern provinces^1^. According to records from the Food and Agriculture Organization (FAO) of the United Nations, China produced 1,939,457 tons of tea in 2013 (http://faostat3.fao.org/download/Q/QC/E). The raw materials for tea products, the buds and leaves of the tea plant, are affected by a number of diseases. Of these diseases, anthracnose caused by *Colletotrichum* spp. is one of the most serious diseases^2^. Leaves infected by *Colletotrichum* generally have water-soaked lesions at the initial stage of the disease. As the disease progresses, the lesions get larger and become necrotic, ultimately leading to serious losses in yield^3^.

*Colletotrichum* Corda is one of the most important fungal genera containing plant pathogens in the world, causing disease in a wide range of hosts^4^,^5^. *Colletotrichum* can inhabit plants as a pathogen, endophyte, epiphyte, or saprobe^5^,^6^,^7^,^8^. Studies of the taxonomy of *Colletotrichum* were once limited to the identification of strains using only inconsistent morphological characteristics and host association^8^,^9^. More recently, the use of morphology coupled with multi-gene molecular phylogeny has developed as an effective strategy for identification and has improved the understanding of *Colletotrichum* taxonomy^4^,^5^,^8^,^9^,^10^. Using this identification strategy, the *Colletotrichum* species were re-classified into 11 important species complexes and some independent species^5^,^11^,^12^,^13^,^14^,^15^,^16^. In addition, several recent studies have used the ApMat DNA locus to accurately and rapidly identify the *Colletotrichum* species^2^,^17^,^18^,^19^.

A single host plant can be infected by multiple *Colletotrichum* species^20^,^21^,^22^,^23^,^24^. Several studies identifying *Colletotrichum* as the causal agent of anthracnose in *Ca. sinensis* have shown that there is remarkable species diversity in *Colletotrichum* present as pathogens or endophytes^3^,^25^,^26^,^27^,^28^. However, these studies only applied either morphology or internal transcribed spacer (ITS) method to identify *Colletotrichum* species, and this approach has been reported inaccurate for interspecific relationship identifications^4^,^8^,^9^,^29^. Furthermore, these studies were also limited by the small area of the investigated regions and lacked information regarding the infected tea plant cultivar. For example, Guo *et al*.^3^ investigated *Colletotrichum* only in yellow mountain tea plants in the Anhui province of China. Liu *et al*.^2^ recently isolated *Colletotrichum* from healthy and diseased tissues of *Camellia* spp. from 7 provinces in China and from 3 other countries. However, their investigations included only partial areas of tea plant cultivation in China. Therefore, further study is necessary to identify *Colletotrichum* on *Ca. sinensis* in China to a species level by their morphological characteristics and multigene phylogenies.

To understand the diversity of *Colletotrichum* species on *Ca. sinensis*, we collected diseased leaves of tea plants from several of the major tea growing regions of China. After isolating and identifying *Colletotrichum* species, we summarized the *Colletotrichum* species associated with *Ca. sinensis* and their geographical distributions in China. We believe these results can provide phytopathologists and plant breeders with a reference for the prevention and control of anthracnose disease.

## Results

### Multilocus-based phylogenetic analysis

We collected 106 isolates of *Colletotrichum* spp. from diseased leaves of *Ca. sinensis* from the main tea growing regions in China and identified them based on phylogeny and morphological characteristics.

**Figure 1:**
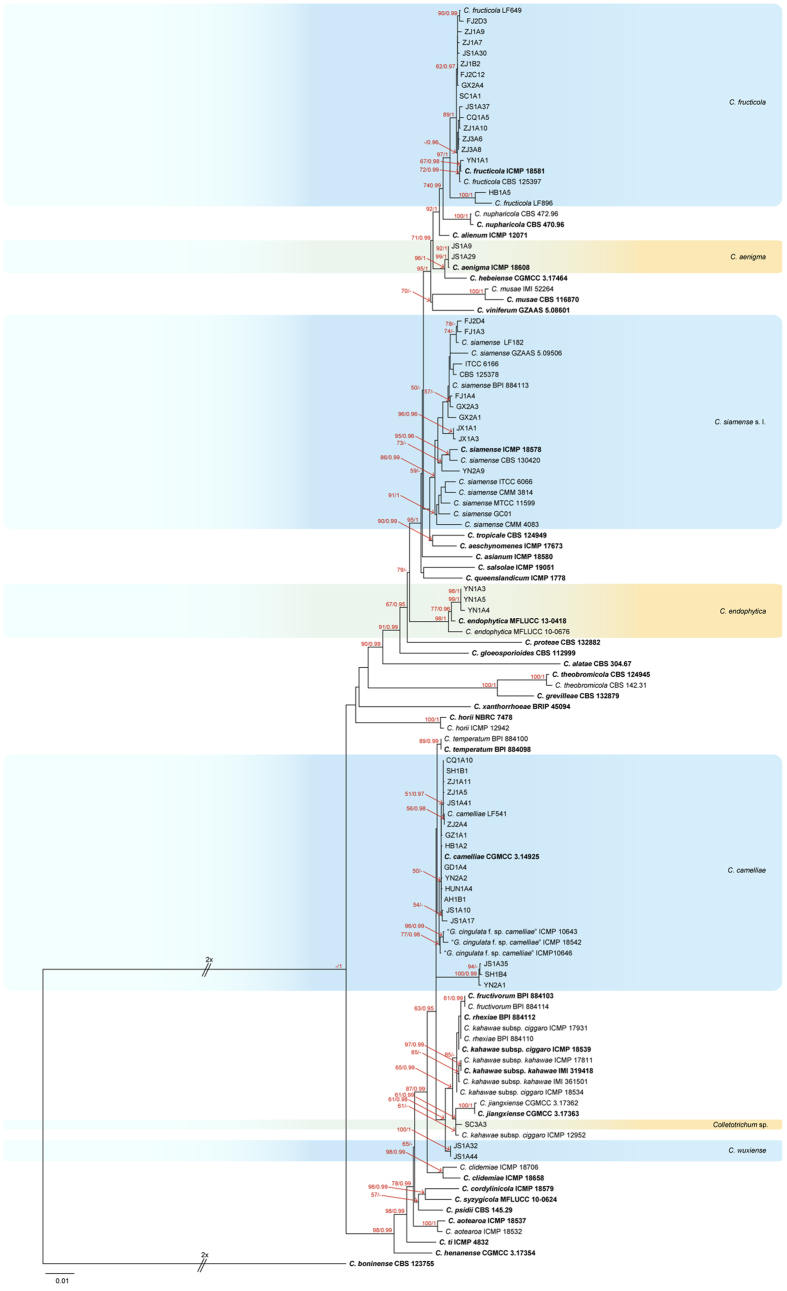
Phylogenetic tree generated by maximum likelihood analysis based on combined ITS, ACT, GAPDH, CAL, CHS-1, TUB2, and GS gene sequences. The tree shows the phylogenetic relationships between *Colletotrichum* species in the *C. gloeosporioides* species complex isolated from *Ca. sinensis*. Bootstrap support values above 50% and Bayesian posterior values above 0.95 are shown at each node (ML/PP). *C. boninense* CBS 123755 is used as outgroup. Branches crossed by diagonal lines are shortened by 50%. Ex-type strains are emphasized in bold.

The phylogram in Fig. 1 shows the identified isolates in the *C. gloeosporioides* species complex. The combined aligned data matrix (ITS, ACT, GAPDH, CAL, CHS-1, TUB2, and GS) contained 152 sequences including the outgroup (*C. boninense* CBS 123755) and 3,879 characters including gaps. These species were determined from 81 isolates from *Ca. sinensis* plants in our study (the partly identical *C. camelliae* and *C. fructicola* isolates were removed from Fig. 1; the complete alignment and tree, shown in Fig. 1c, was available from TreeBASE). Of the 81 isolates, 2 clustered with *C. aenigma* ex-type culture, 33 clustered with *C. camelliae*, 3 clustered with *C. endophytica*, 34 clustered with *C. fructicola*, 8 clustered with *C. siamense*, 1 (SC3A3) was indistinguishable and named *Colletotrichum* sp., and 2 (JS1A32, JS1A44) did not cluster with any known species and formed a distinct clade with a high bootstrap support/posterior probability value (61/0.98).

**Figure 2:**
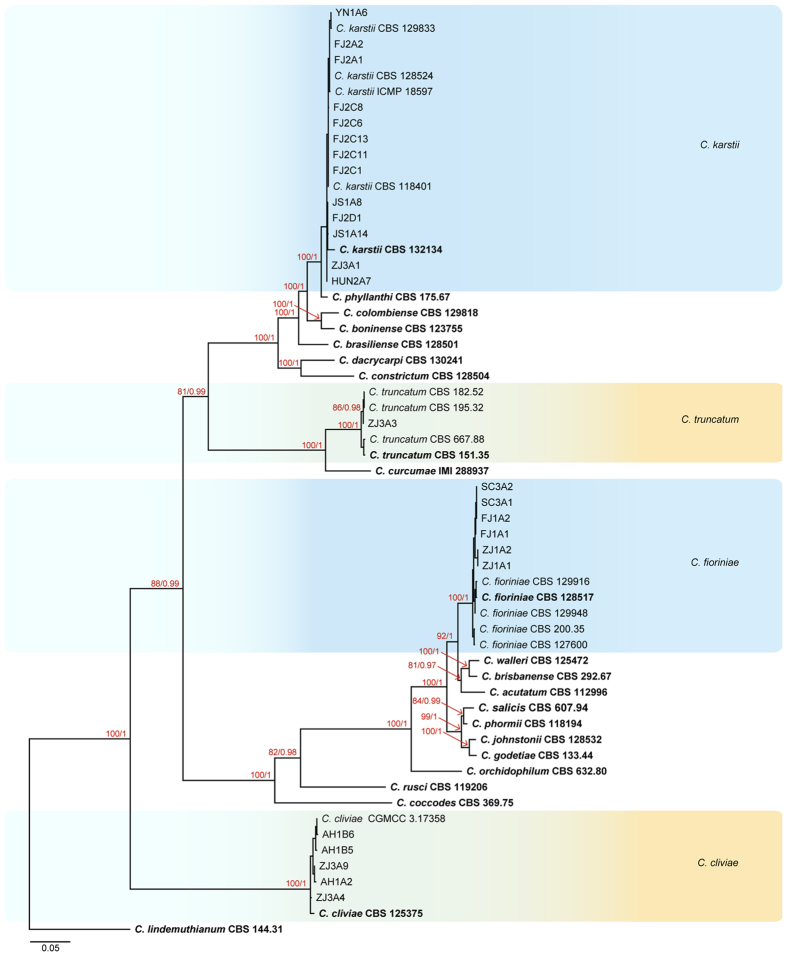
Phylogenetic tree generated by maximum likelihood analysis based on combined ITS, ACT, GAPDH, TUB2, and CHS-1 gene sequences. The tree shows the phylogenetic relationships between *Colletotrichum* species outside of the *C. gloeosporioides* species complex isolated from *Ca. sinensis*. Bootstrap support values above 50% and Bayesian posterior values above 0.95 are shown at each node (ML/PP). *C. lindemuthianum* CBS 144.31 is used as outgroup. Ex-type strains are emphasized in bold.

The phylogram in Fig. 2 shows the isolates of *Colletotrichum* that were not in the *C. gloeosporioides* species complex. The combined aligned data matrix (ITS, ACT, GAPDH, TUB2, and CHS-1) contained 59 sequences including the outgroup (*C. lindemuthianum* CBS 144.31) and 1,928 characters including gaps. These data were determined from 25 isolates from *Ca. sinensis* in our study. The maximum likelihood tree (tree topology) had bootstrap support values greater than 50% and Bayesian posterior probability values of ≥0.95. The 25 isolates were grouped into 4 subclades: *C. cliviae*, *C. fioriniae, C. karstii*, and *C. truncatum*. Thirteen isolates clustered with the ex-type strain of *C. karstii* CORCG6, 1 isolate clustered with *C. truncatum*, 6 isolates clustered with *C. fioriniae*, and 5 isolates clustered with *C. cliviae*.

### ApMat & GS-based phylogenetic analysis of the *C. gloeosporioides* species complex

**Figure 3:**
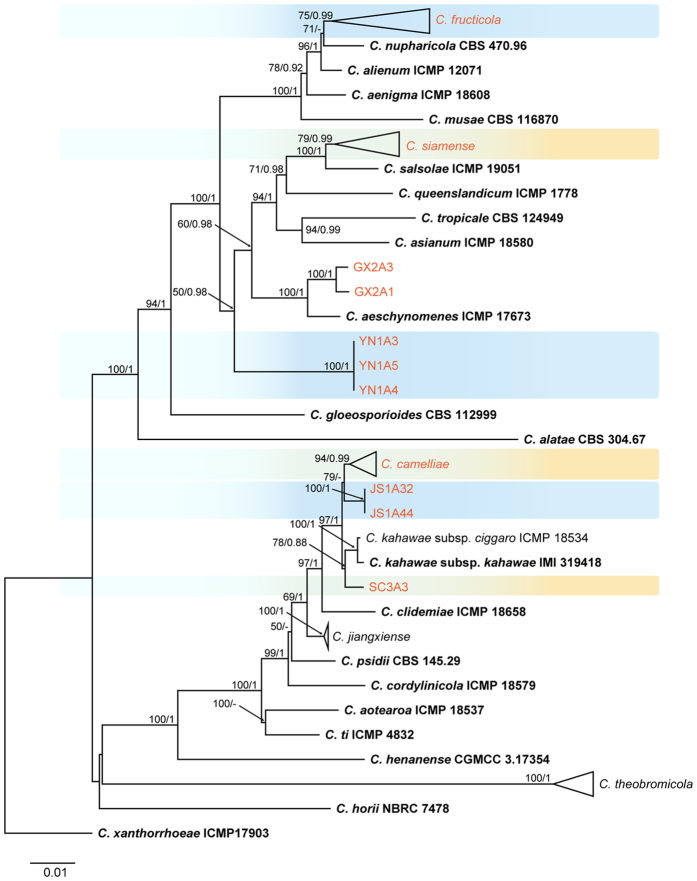
Phylogenetic tree generated by maximum likelihood analysis based on ApMat and GS sequences. Bootstrap support values above 50% and Bayesian posterior values above 0.85 are shown at each node (ML/PP). *C. xanthprrhpeae* ICMP 17903 is used as outgroup. Ex-type strains are emphasized in bold.

Forty-seven strains of the *C. gloeosporioides* species complex isolated from tea plants and 35 reported strains were used for phylogenetic tree construction (rooted with *C. xanthorrhoeae*) based on ApMat and GS sequences. The dataset comprised 1,829 characters with alignment gaps (Fig. 3). All species could be separated with high support values, followed by multi-locus phylogenetic tree analysis, with the exception of isolates GX2A1 and GX2A3. These 2 isolates belonged to *C. siamense* rather than *C. aeschynomenes* (Fig. 1). Sequence alignment of ApMat and GS showed that the isolates GX2A1 and GX2A3 differed from *C. aeschynomenes* ICMP 17673 by only 5 bp in ApMat but by 19 bp in GS. The strain SC3A3 clustered with *C. kahawae* sensu lato.

### Pairwise homoplasy index (PHI) test

**Figure 4:**
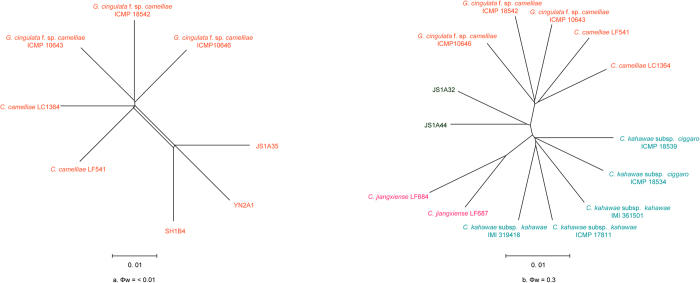
The results of pairwise homoplasy index (PHI) test of closely related species using both LogDet transformation and splits decomposition. PHI test results (Φw) <0.05 indicate significant recombination within the dataset.

Application of the PHI test to the concatenated 6-locus sequences (ITS, ACT, GAPDH, CAL, TUB2, and GS) revealed the recombination level within phylogenetically related species (Φw < 0.05). Significant recombination events were detected between *C. camelliae* and three closely related strains (JS1A35, SH1B4, and YN2A1) (Φw = <0.01) (Fig. 4a). No significant recombination events were observed between *C. wuxiense* and phylogenetically related species such as *C. camelliae*, *C. jiangxiense*, and *C. kahawae* s. l. (Φw = 0.3) (Fig. 4b).

### Pathogenicity tests

Pathogenicity tests showed that *C. aenigma* CGMCC 3.17883, *C. camelliae* CGMCC 3.17884, and *C. endophytica* CGMCC 3.17887 have the typical brown lesions of anthracnose disease around wounded areas. Conidia from wounds were re-isolated and cultured on potato dextrose agar (PDA), which showed pathogenesis analogous to that of infected strains. However, wounded leaves of *Ca. sinensis* cv. *Longjing 43* did not have obvious disease spots after inoculation with *Colletotrichum* sp. CGMCC 3.17890, *C. siamense* CGMCC 3.17892, or *C. wuxiense* CGMCC 3.17894 (see Supplementary Figs S4l, S9m and S10m). A possible explanation for the few disease spots caused by these strains is that the strains have weak virulence or lack necessary pathogenesis genes for *Longjing 43* (the gene-for-gene hypothesis)^30^,^31^.

### Taxonomy

Based on their multi-locus phylogeny and morphological characteristics, the 106 isolates from *Ca. sinensis* were identified as 11 species of *Colletotrichum* (Figs 1, 2, 3), including 1 new species (named *C. wuxiense*), 3 new record species (*C. aenigma*, *C. endophytica*, and *C. truncatum*), 6 previously described species (*C. camelliae*, *C. cliviae*, *C. fioriniae*, *C. fructicola*, *C. karstii*, and *C. siamense*), and 1 indistinguishable strain (described as *Colletotrichum* sp.).

***Colletotrichum aenigma*** B.S. Weir & P.R. Johnston, Studies in Mycology 73: 135. 2012. See Supplementary Fig. S1.

Description: Colonies on PDA were flat with entire edges, aerial mycelium sparse, cottony, pale white, scattered acervuli with yellow conidial mass near the center, pale white on reverse, had a growth rate of 11.4–12.3 mm per day at 25 °C after 5 days. Sexual morph was observed on PDA agar after 7 days. *Ascomata* globose, brown to dark brown, covered by sparse and white aerial mycelium, outer walls composed of dark brown verruculose angular cells. *Asci* clavate, falciform, 64.0–111.0 × 12.0–17.0 μm, n = 8, 8-spored. *Ascospores* arranged biseriately, hyaline, aseptate, smooth, ellipsoidal or ovoid, 13.4–16.6 × 5.9–8.3 μm, av ± SE = 14.8 ± 1.1–6.7 ± 0.8 μm, L/W ratio = 2.2, n = 30. Asexual morph was observed, *Chlamydospores* were not observed. *Conidiomata* acervular. *Setae* observed, dark brown, smooth, 2-septate, 112.0–123.0 μm long, the base rounded, 1.6–4.8 μm diameter, tip somewhat acute. *Conidiophores* and *conidiogenous* cells were not observed. *Conidia* hyaline, aseptate, smooth, cylindric with broadly rounded ends, 13.2–20.0 × 5.2–7.3 μm, av ± SE = 17.2 ± 1.5–6.1 ± 0.5 μm, L/W ratio = 2.8, n = 30. *Appressoria* subglobose or with a few broad lobes, brown, branched, 8.6–16.6 × 5.2–10.6 μm, av ± SE = 11.8 ± 2.0–7.9 ± 1.2 μm, L/W ratio = 1.5, n = 30.

Materials examined: CHINA, Jiangsu Province, Wuxi City, from diseased leaves of *Ca. sinensis*, 20 Aug 2014. Y.C. Wang, culture JS1A9; ibid., culture CGMCC 3.17883 = JS1A29.

Notes: *Colletotrichum aenigma* was first reported on *Persea americana* from Israel and has been subsequently reported on *Pyrus pyrifolia* from Japan^13^, *Pyrus communis*, *Citrus sinensis*, and *Olea europaea* from Italy^32^, *Hylocereus undatus* from Thailand^33^, *Poplar* sp. from China^22^,^34^, and *Vitis vinifera* from China^24^. However, none of these studies described the sexual morph and setae of this species. This is the first report of *C. aenigma* associated with anthracnose of *Ca. sinensis* in China and the first description of the morphological characteristics of its sexual morph and seta.

***Colletotrichum camelliae*** Massee, Bull. Misc. Inform. Kew. 1899: 91. 1899. See Supplementary Fig. S2.

Description: Colonies on PDA raised centers, aerial mycelium dense, cottony, iron-grey. *Chlamydospores* not observed, reverse buff, a growth rate of 11.9–12.9 mm per day at 25 °C after 5 days. Sexual morph was not observed. *Chlamydospores* black, hidden in medium. Only one acervular was observed, *conidiophores* and *setae* either directly formed from hyphae or on a cushion of roundish hyaline cells. *Setae* dark brown, smooth-walled, 1–2 septate, 56.3 μm long, base inflated or cylindrical, 2.0–5.4 μm diameter, tip more or less acute. *Conidiophores* abundant, hyaline, smooth, septate, branched. *Conidiogenous* cells hyaline, cylindrical, 8.1–16.2 × 4.9–6.2 μm. *Conidia* hyaline, aseptate, smooth, cylindrical with obtuse ends or narrowed towards the base, 14.4–18.2 × 5.6–7.4 μm, av ± SE = 16.5 ± 1.0 × 6.5 ± 0.4 μm, L/W ratio = 2.5, n = 30. *Appressoria* irregularly shaped, fusiform, crenate, lobed, brown to dark brown, branched, 8.0–20.3 × 5.2–12.7 μm, av ± SE = 12.3 ± 2.5 × 8.5 ± 1.7 μm, L/W ratio = 1.4, n = 30.

Materials examined: CHINA, Chongqing City, Yongchuan County, from diseased leaves of *Ca. sinensis*, 20 Jul. 2014, Y.C. Wang, culture CQ1A10. Jiangsu Province, Wuxi City, from diseased leaves of *Ca. sinensis*, 20 Jul. 2014, Y.C. Wang; ibid., culture CGMCC 3.17884 = JS1A35. Shaanxi Province, Hanzhong City, Xixiang County, from diseased leaves of *Ca. sinensis* cv. *Fuding Dabaicha*, 30 Jun. 2014, Y.C. Wang, culture SH1B4. Yunan Province, Puer City, Menghai County, from diseased leaves of *Ca. sinensis*, 10 Sept. 2014, Y.C. Wang, culture YN2A1.

Notes: The morphological characteristics of *Colletotrichum camelliae* were systematically described in detail based on methodology used in previous studies^2^. Although *C. camelliae* had been described previously as the dominant *Colletotrichum* species on *Camellia* plants in China^2^,^13^,^18^, the characteristics of its seta had not been investigated. In the present study, the morphological characteristics of setae were described. Interestingly, 3 strains clustered with *C. camelliae* LF789 and formed a distinct subclade as shown in Fig. 1c. However, ApMat and GS-based phylogenetic analysis showed that these 3 strains were distinctly clustered with *C. camelliae* (Fig. 3). The PHI test also revealed significant genetic recombination levels between these 3 strains and *C. camelliae*, suggesting that they are conspecific. In addition, conidia and appressorium dimensions of the 2 strains from Jiangsu and Yunnan Province (JS1A35, conida: 14.4–18.2 × 5.6–7.4 μm, av = 16.5 × 6.5 μm, appressoria: 8.0–20.3 × 5.2–12.7, av = 12.3 × 8.5 μm; YN2A1, conida: 15.7–20.1 × 4.9–6.8 μm, av = 18.1 × 5.8 μm, appressoria: 6.1–18.0 × 6.3–11.8, av = 11.3 × 8.6 μm) were in accordance with *C. camelliae* ex-type culture, while the mean values were larger than those of ex-type culture (GMCC 3.14925, conida: 9.0–25 × 3.5–7.5 μm, av = 15.5 × 5.0 μm, appressoria: 6.5–13.5 × 5.0–10.5 μm, av = 10.0 × 7.5 μm).

***Colletotrichum cliviae*** Y.L. Yang, Zuo Y. Liu, K.D. Hyde & L. Cai, Fungal Diversity 39: 133. 2009.

Description and illustrations: see Yang *et al*.^35^ and Liu *et al*.^2^.

Materials examined: CHINA, Anhui Province, Huangshan City, Qimen County, from diseased leaves of *Ca. sinensis* cv. *Keemenzhong*, 27 Aug. 2014, Y.C. Wang, culture AH1A2; ibid., culture CGMCC 3.17885 = AH1B5; ibid., culture AH1B6. Zhejiang Province, Hangzhou city, from diseased leaves of *Ca. sinensis* cv. *Longjing 43*, 8 Jun. 2015, Y.C. Wang, culture ZJ3A4; ibid., culture ZJ3A9.

Notes: *Colletotrichum cliviae* was identified previously on a healthy tea leaf from Guilin, Guangxi Province, China^2^. In the present study, 5 strains of pathogenic *C. cliviae* were isolated from *Ca. sinensis* cv*. Keemenzhong* from Anhui and cv. *Longjing 43* from Zhejiang Province, China.

***Colletotrichum endophytica*** Manamgoda, Udayanga, L. Cai & K.D. Hyde, Fungal Diversity 61: 107–115. 2013. See Supplementary Fig. S3.

Description: Colonies on PDA flat with entire edge, aerial mycelium sparse, pale white, scattered numerous acervuli with orange conidial mass, reverse scattered orange colored pigmentation around the center and white aerial mycelium near the margin, a growth rate of 12.6–13.6 mm per day at 25 °C after 5 days. Sexual morph was not observed. *Conidiomata* acervular. *Chlamydospores* not observed. *Setae* brown, 2–3 septate, 73.6–105.5 μm long, base more or less inflated, 1.8–3.3 μm diameter, tip usually acute. *Conidiophores* abundant, hyaline, smooth, septate, branched. *Conidiogenous* cells hyaline, cylindrical, ampulliform, 11.1–15.1 × 3.6–4.9 μm. *Conidia* hyaline, aseptate, smooth, cylindrical, 16.2–19.9 × 4.2–6.0 μm, av ± SE = 18.4 ± 0.9 × 5.0 ± 0.5 μm, L/W ratio = 3.7, n = 30. *Appressoria* irregularly shaped, unlobed or slightly lobed, brown to dark brown, unbranched, 7.9–18.0 × 4.6–11.6 μm, av ± SE = 12.6 ± 2.8 × 7.8 ± 1.5 μm, L/W ratio = 1.6, n = 30.

Materials examined: CHINA, Yunnan Province, Lincang City, from diseased leaves of *Ca. sinensis* cv. *Menku*, 17 Jul. 2014, Y.C. Wang, culture CGMCC 3.17886 = YN1A3; ibid., culture CGMCC 3.17887 = YN1A4; ibid., culture YN1A5.

Notes: *Colletotrichum endophytica* had been found only as an endophyte on *Pennisetum purpureum*^6^ and on unknown wild fruit from Thailand^36^. In the present study, 3 pathogenic strains were isolated from diseased leaves of *Ca. sinensis.* This is the first report of *C. endophytica* causing anthracnose in *Ca. sinensis* and the first report of *C. endophytica* in China. Setae of *C. endophytica* are observed and described in this study for the first time.

***Colletotrichum fioriniae*** (Marcelino & Gouli) R.G. Shivas & Y.P. Tan, Fungal Diversity 39: 117. 2009.

Description and illustrations: see Damm *et al*.^11^.

Materials examined: CHINA, Sichuan Province, Meishan City, Hongya County, from diseased leaves of *Ca. sinensis* cv. *Mingshanzao 132*, 10 Sept. 2014, Y.C. Wang, culture CGMCC 3.17888 = SC3A2. Zhejiang Province, Hangzhou City, on *Ca. sinensis* cv. *Longjing 43*, 20 Jul. 2014, Y.C. Wang, culture ZJ1A1.

Notes: *Colletotrichum fioriniae* has been reported on various host plants, including *Camellia* plants in Kunming, Yunnan Province, and Fuzhou, Fujian Province, China^11^,^28^. In the present study, the species were isolated from *Ca. sinensis* cv. *Fuding Dahaocha* and cv. *Huangdan* from Fuzhou, Fujian Province, from cv. *Mingshanzao 132* from Meishan, Sichuan Province, and from cv. *Longjing 43* from Hangzhou, Zhejiang Province, China.

***Colletotrichum fructicola*** Prihastuti, L. Cai & K.D. Hyde, Fungal Diversity 39: 158. 2009.

Description and illustrations: see Prihastuti *et al*.^23^ and Liu *et al*.^2^.

Materials examined: CHINA, Chongqing City, Yongchuan County, from diseased leaves of *Ca. sinensis*, 20 Jul. 2014, Y.C. Wang, culture CGMCC 3.17889 = CQ1A5. Hubei Province, Enshi City, from diseased leaves of *Ca. sinensis*, 18 Jul. 2014, Y.C. Wang, culture HB1A5. Sichuan Province, Yibin City, Jiangan County, from diseased leaves of *Ca. sinensis* cv. *Zhongcha 302*, 16 Jul. 2014, Y.C. Wang, culture SC1A1. Zhjiang Province, Hangzhou City, from diseased leaves of *Ca. sinensis* cv. *Longjing 43*, 20 Jul. 2014, Y.C. Wang, culture ZJ3A6.

Notes: *Colletotrichum fructicola* was first found in coffee berries and has since been found to cause disease in several plants^23^. It was recently reported to cause anthracnose in *Ca. sinensis* in 5 Provinces in China^2^,^28^. In the present study, the species was found on multiple *Ca. sinensis* cultivars from almost all of the main tea growing areas in China.

***Colletotrichum karstii*** Y.L. Yang, Z.Y.Liu, K.D. Hyde & L.Cai, Cryptogamie Mycologie 32: 241. 2011.

Description and illustrations: see Yang *et al*.^37^ and Damm *et al*.^12^.

Materials examined: CHINA, Fujian Province, Quanzhou City, Anxi County, from diseased leaves of *Ca. sinensis* cv. *Huangjingui*, 21 Jul. 2014, Y.C. Wang, culture FJ2A1; ibid., from diseased leaves of *Ca. sinensis* cv. *Maoxie*, 21 Jul. 2014, Y.C. Wang, culture FJ2C6. Hunan Province, Changsha City, from diseased leaves of *Ca. sinensis*, 15 Sept. 2014, Y.C. Wang, culture HUN2A7. Jiangsu Province, Wuxi City, from diseased leaves of *Ca. sinensis*, 20 Aug. 2014, Y.C. Wang, culture JS1A8. Yunnan Province, Lincang City, from diseased leaves of *Ca. sinensis* cv. *Menku*, 17 Jul. 2014, Y.C. Wang, culture YN1A6.

Notes: *Colletotrichum karstii* is the most common *Colletotrichum* and is present in a wide range of hosts. It was previously reported to be pathogenic to *Ca. sinensis* from Fujian^28^ and Zhejiang provinces^2^. In this study, species were isolated from the Fujian, Zhejiang, Yunnan, Jiangsu, and Hunan Provinces of China.

***Colletotrichum*** **sp**. See Supplementary Fig. S4.

Description: Colonies on PDA raised centers, aerial mycelium dense, cottony, grey, reverse olivaceous to grey colored pigmentation, a growth rate of 7.3–12.4 mm per day at 25 °C after 5 days. Sexual morph was not observed. *Chlamydospores* black, among the aerial mycelium. *Conidiomata*, *seta* and *Conidiophores* not observed. *Conidia* hyaline, aseptate, smooth, cylindrical, clavate, straight, 14.5–20.7 × 5.2–7.5 μm, av ± SE = 17.3 ± 1.7 × 6.1 ± 0.5 μm, L/W ratio = 2.8, n = 30. *Appressoria* irregularly shaped, unlobed or slightly lobed, brown to dark brown, unbranched, 8.9–14.7 × 6.6–12.2 μm, av ± SE = 11.6 ± 1.6 × 8.9 ± 1.5, L/W ratio = 1.3, n = 30.

Materials examined: CHINA, Sichuan Province, Meishan City, Hongya County, from diseased leaves of *Ca. sinensis* cv. *Mingshanzao 132*, 10 Sept. 2014, Y.C. Wang, culture CGMCC 3.17890 = SC3A3.

Notes: In the phylogenetic tree, strain SC3A3 appears as a sister clade between *C. kahawae* s. l. and *C. jiangxiense* (Fig. 1). Its sequences (ITS, ACT, GAPDH, CAL, CHS-1, and TUB2) were identical to those of ICMP 12952, but its GS sequence differed by 8 bp. The GS sequence of the SC3A3 strain also differed from that of the ex-type culture of *C. jiangxiense* (CGMCC 3.17363) by 25 bp and 1 bp indel. Conidia of strain SC3A3 (av = 17.3 × 6.1 μm) were similar to the extype culture of *C. kahawae* subsp. *ciggaro* (ICMP 18539, av = 17.8 × 5.1) but larger than those of the ex-type culture of *C. jiangxiense* (CGMCC 3.17363, av = 15.2 × 5.2 μm).

***Colletotrichum siamense*** Prihastuti, L. Cai & K.D. Hyde, Fungal Diversity 39: 158. 2009. See Supplementary Fig. S5.

Description: Colonies on PDA raised centers, aerial mycelium dense, pale white, scattered little acervuli with orange conidial mass around the center, reverse viridescent to pale white, a growth rate of 12.4–13.5 mm per day at 25 °C after 5 days. Sexual morph was not observed. *Conidiomata* acervular. *Chlamydospores* black, hidden in medium. *Setae* brown, 4 septate, 69.0 μm long, base more or less inflated, 1.5–3.3 μm diameter, tip usually acute. *Conidiophores* formed on a cushion of roundish with medium brown cells, hyaline, smooth, aseptate, branched. *Conidiogenous* cells hyaline, cylindrical, ampulliform, 10.7–16.8 × 5.1–5.9 μm. *Conidia* hyaline, aseptate, smooth, cylindrical, straight or slight curved, 16.4–19.4 × 4.4–6.1 μm, av ± SE = 16.8 ± 1.2 × 5.3 ± 0.4 μm, L/W ratio = 3.1, n = 30. *Appressoria* circular or ellipsoidal, brown, branched, 6.9–15.6 × 6.1–10.1 μm, av ± SE = 10.1 ± 2.0 × 8.0 ± 1.2 μm, L/W ratio = 1.3, n = 30.

Materials examined: CHINA, Fujian Province, Fuzhou City, from diseased leaves of *Ca. sinensis* cv. *Fujian Shuixian*, 20 Jul. 2014, Y.C. Wang, culture FJ1A3; ibid., from diseased leaves of *Ca. sinensis* cv. *Fujian Shuixian*, 20 Jul. 2014, Y.C. Wang, culture FJ1A4; ibid., Quanzhou City, Anxi County, from diseased leaves of *Ca. sinensis* cv. *Tie guanyin*, 21 Jul. 2014, Y.C. Wang, culture FJ2D4. Guangxi Province, Guilin City, from diseased leaves of *Ca. sinensis* cv. *Jiukang*, 29 Aug. 2014, Y.C. Wang, culture CGMCC 3.17891 = GX2A1; ibid., CGMCC 3.17892 = GX2A3. Jiangxi Province, Nanchang City, from diseased leaves of *Ca. sinensis* cv. *Fuding Dabaicha*, 2 Sept. 2014, Y.C. Wang, culture JX1A1; ibid., JX1A3. Yunan Province, Puer City, Menghai County, from diseased leaves of *Ca. sinensis*, 10 Sept. 2014, Y.C. Wang, culture YN2A9.

Notes: *Colletotrichum siamense* was originally found on coffee berries from Thailand, has been found on various hosts, and now is considered a biologically and geographically diverse species^13^,^23^,^35^,^38^. A previous study reported that *C. siamense* caused anthracnose of several varieties of *Ca. sinensis* from many regions of China^28^. The species can be distinguished from other species in the *C. gloeosporioides* species complex through analysis of concatenated ApMat and GS sequences^2^,^17^,^18^. In the present study, multi-locus phylogenetic trees and morphological characterization were used to identify strains GX2A1 and GX2A3 as *C. siamense* (Fig. 1). However, in a phylogenetic tree constructed from ApMat and GS sequences, these 2 strains clustered with the ex-type strain of *C. aeschynomenes* ICMP 17673 rather than with *C. siamense s. l.* The ApMat sequences of the 2 strains (GX2A1 and GX2A3) were identical to those of ICMP 17673 with the exception of just 5 bp. Therefore, analysis of a single gene sequence or analysis of ApMat and GS gene sequences together were not sufficient to accurately separate the *C. siamense.*

***Colletotrichum truncatum*** (Schwein.) Andrus & W.D. Moore, Phytopathology 25: 122. 1935.

Description and illustrations: see Andrus & Moore^39^ and Damm *et al*.^16^.

Materials examined: CHINA, Zhejiang Province, Hangzhou City, from diseased leaves of *Ca. sinensis* cv. *Longjing 43*. 10 Aug. 2015, Y.C. Wang, culture CGMCC 3.17893 = ZJ3A3.

Notes: *Colletotrichum truncatum* is present in a wide range of hosts^16^. This is the first report showing that this species can infect *Ca. sinensis* in China.

**Figure 5:**
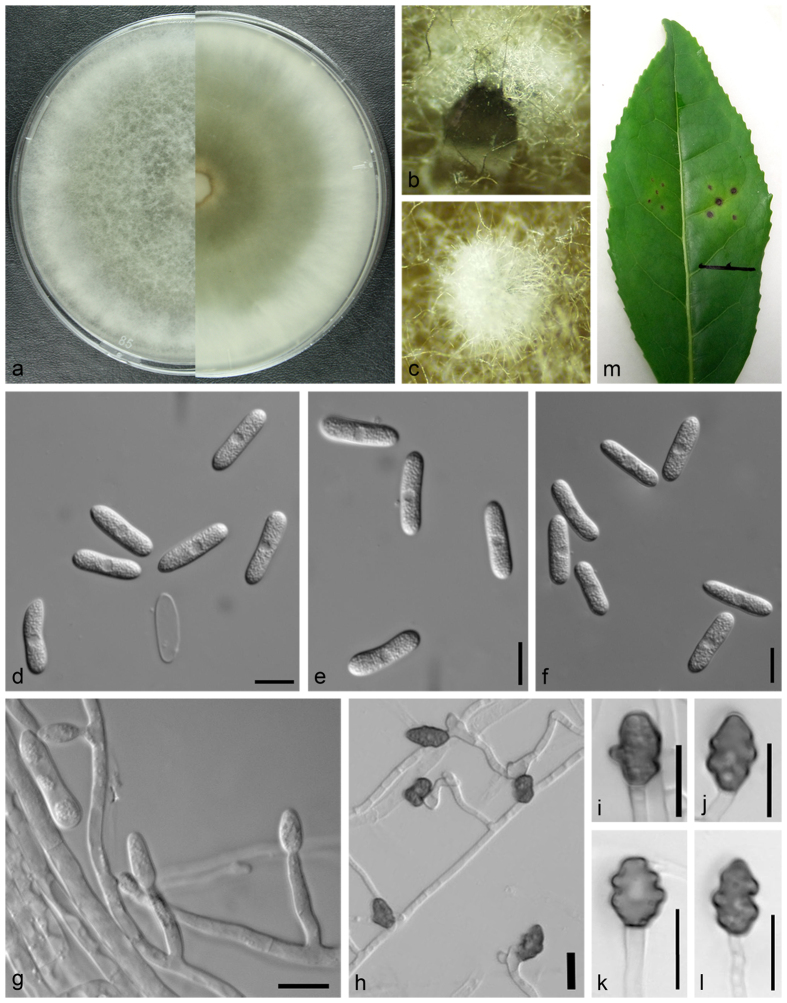
*Colletotrichum wuxiense* (from strain JS1A32). (**a**) colony, upper and reverse; (**b–c**) chlamydospores; (**d**–**f**) conidia; (**g**) conidiophores; (**h–l**) appressoria; m. induced symptoms on leaf after 14 days. (**a–g**) from PDA agar medium in 7 days; (**h–l**) from SNA agar medium. Scale: 10 μm.

***Colletotrichum wuxiense*** Y.C. Wang, X.C. Wang & Y.J. Yang. sp. nov. Fig. 5.

MycoBank: MB 816242.

Etymology: This fungus was first collected from Wuxi city, Jiangsu province in China.

Description: Colonies on PDA were flat with entire edge, aerial mycelium dense, cottony, white, reverse olivaceous colored pigmentation around the center to white near the margin, a growth rate of 13.4–14.1 mm per day at 25 °C after 5 days. Sexual morph was not observed. *Chlamydospores* black, among the aerial mycelium, *conidiomata* and *seta* not observed. *Conidiophores* rare, formed directly on aerial mycelium, hyaline, aseptate, unbranched. *Conidiogenous* cells hyaline, cylindrical. *Conidia* hyaline, aseptate, smooth, cylindrical with obtuse ends or narrowed towards the base, straight or slightly curved, 16.5–23.1 × 4.6–6.7 μm, av ± SE = 19.0 ± 1.4 × 5.6 ± 0.5 μm, L/W ratio = 3.8, n = 30. *Appressoria* elliptic to subfusoid, deeply lobed, brown, unbranched, 5.2–14.6 × 5.8–10.5 μm, av ± SE = 10.2 ± 2.1 × 7.7 ± 1.1 μm, L/W ratio = 1.3, n = 30.

Materials examined: CHINA, Jiangsu Province, Wuxi City, from diseased leaves of *Ca. sinensis*, 20 Aug. 2014, Y.C. Wang, Holotype HMAS 246948, culture ex-type CGMCC 3.17894 = JS1A32; ibid., culture JS1A44.

Notes: Multi-locus (ITS, ACT, GAPDH, CAL, CHS-1, TUB2, and GS) phylogenetic tree analysis showed that C*olletotrichum wuxiense* is a sister clade of the closely related *C. jiangxiense* and *C. kahawae* s. l., and it clustered with *C. camelliae* (Fig. 1). *C. wuxiense* can be distinguished from these other species by the morphological features of its conidia, which are larger than those of these similar species^2^,^13^ and are slightly curved. In addition, *C. wuxiense* can be directly separated from other species of the *C. gloeosporioides* species complex using its concatenated ApMat and GS gene tree (Fig. 3). A PHI test also showed that no significant recombination events between *C. wuxiense* and closely phylogenetically related species occurred.

### Prevalence of *Colletotrichum* species

**Figure 6:**
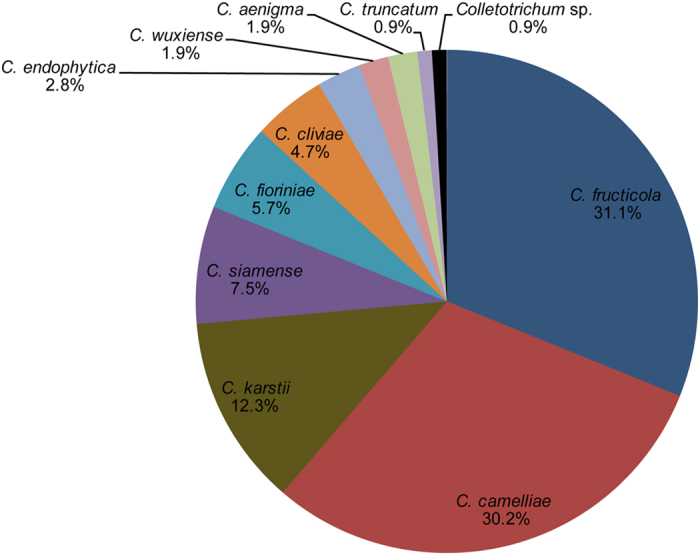
Isolation Rate (IR%) of *Colletotrichum* species that were isolated from tea plants from China. The pie chart is created by Microsoft Excel 2010 (https://products.office.com/en-us/buy/using-your-office-2010-product-key-card). Only data from our study were used.

Of the 106 isolates of *Colletotrichum*, 33 isolates of *C. camelliae* were isolated from 14 provinces/cities, and 34 isolates of *C. fructicola* were isolated from 11 provinces/cities (Table 1). Approximately 61.3% of the total isolates were harvested from tea-producing areas of China (Table 1, Fig. 6). These results suggest that *C. camelliae* and *C. fructicola* are the dominant species causing anthracnose of *Ca. sinensis*. Moreover, 7 of 11 species belonged to the *C. gloeosporioides* species complex (Figs 1 and 2, Table 1, Supplementary Table S1).

## Discussion

In this study, a total of 106 *Colletotrichum* isolates obtained from the diseased leaves of tea cultivars in China were identified as 11 species, of which 9 had been described previously, 1 was identified as a new species, and 1 was unidentifiable.

*C. gloeosporioides* was previously considered the dominant *Colletotrichum* species on tea plants in China^3^. In our study, *C. camelliae* and *C. fructicola* were the most prevalent species in China. These results were similar to those of Liu, *et al*.^2^. *C. camelliae* were collected from 14 out of 15 provinces of China (Table 1, Fig. 6, Supplementary Table S1). Nevertheless, 3 strains of *C. camelliae* (YN2A1, JS1A35, and SH1B4) formed a separate clade and were close to *C. camelliae* (CGMCC 3.14925) in the phylogenetic tree (Fig. 1). Moreover, conidiophores and setae of strain JS1A35 were directly produced from hyphae or on a cushion of roundish hyaline cells, rather than on aerial mycelium (CGMCC 3.14925). In addition, we also observed intraspecific differences in the colonial morphology and growth rate of *C. camelliae* isolates. Therefore, the genetic differentiation among the above isolates with different geographic distribution and morphology should be further clarified. *C. fructicola*, which was obtained from 11 provinces across China, was the second most prevalent species in our study (Table 1, Fig. 6, Supplementary Table S1). Liu^28^ reported that this species could infect several varieties of *Ca. sinensis* in the Fujian province of China. Liu, *et al*.^2^ further corroborated this finding using 32 isolates. Hence, we believe that the *C. fructicola* was a latent dominant species.

Research on new record species of microorganisms in hosts can provide helpful information for understanding the interactions between hosts and microorganisms as well as their geographical distribution. *Colletotrichum* species could switch their lifestyle from endophytic to pathogenic, for which both internal and external environmental factors play important roles^5^,^40^. In our study, 3 new record species from *Ca. sinensis* were reported for the first time, including *C. aenigma*, *C. endophytica* and *C. truncatum*. *C. endophytica* was described as an endophyte or saprobe in *Pennisetum purpureum* and an unknown wild fruit^6^,^36^. Interestingly, our study showed that *C. endophytica* also can be a pathogen that infects *Ca. sinensis* (see Supplementary Fig. S3j). We speculated that there is a specific interaction between *Ca. sinesis* and *C. endophytica*^31^,^41^.

We collected a strain (SC3A3) that was not well distinguished and we classified it as *Colletotrichum* sp. using the multi-locus phylogenetic tree (Fig. 1). Its morphological characteristics were more similar to *C. kahawae* subsp. *ciggaro* than to *C. jiangxiense* (see Supplementary Fig. S4). Therefore, further studies are required to clarify the phylogenetic relationships among these species.

Previous studies indicated that *Ca. sinensis* can harbor various *Colletotrichum* species. *C. acutatum*^25^ and *C. gloeosporioides*^42^ were generally considered as dominantly endophytic. A few other species were considered as pathogenic or potentially pathogenic on *Camellia*, such as *C. lupini*, *C. acutatum*, *C. carveri*, *C. coccodes* and *C. queenslandicum*^11^,^13^. In this study, we collected the common species, such as *C. cliviae, C. fioriniae, C. karstii,* and *C. siamense*, as well as a novel species that was named *C. wuxiense.* However, *C. acutatum* and *C. gloeosporioides* were not the dominant species. Similar results were also reported by Liu, *et al*.^2^ in the *Colletotrichum* classification study. These differences in dominant species identification may be caused by the variation of sampling range between studies.

In conclusion, we investigated the diversity of *Colletotrichum* species in tea plants in China and identified 11 species including 1 novel species. Moreover, we found that *C. camelliae* and *C. fructicola* are the dominant species in *Ca. sinensis*. Unfortunately, our study failed to characterize endogenetic *Colletotrichum* species due to the lack of healthy tissue collected from tea plants. In future studies, we will isolate endophytic *Colletotrichum* species from healthy tissues of tea plants and elucidate their geographical distribution, the evolutionary relationship between *Colletotrichum* and *Ca. sinensis*, and the differences in intraspecific virulence and morphology of *C. camelliae*.

## Materials and Methods

### Collection and isolation

Diseased leaves with visible anthracnose symptoms were collected from the following 15 provinces or cities of China: the provinces of Anhui, Fujian, Guangdong, Guangxi, Guizhou, Henan, Hunan, Hubei, Jiangsu, Jiangxi, Shaanxi, Sichuan, Yunnan, and Zhejiang and the city of Chongqing. Five randomly selected diseased leaves were sampled from each cultivar and region. *Colletotrichum* species were isolated by a single spore isolation technique as described by Cai, *et al*.^4^. Spore masses were picked off with a sterilized wire loop and suspended in sterilized water. The spore suspension was diluted to a reasonable concentration and spread onto the surface of PDA, followed by an incubation overnight at 25 °C. Single germinating spores were picked up with a sterilized needle and transferred to a new PDA plate.

### DNA extraction, PCR amplification, and sequencing

Fungal isolates were grown for 5–7 days on PDA. Mycelia were collected in a sterile centrifuge tube and stored at −80 °C for DNA extraction. Total genomic DNA of the isolate was extracted using a Ezup Column Fungi Genomic DNA Purification Kit (Sangon Biotech Shanghai Company Limited, Shanghai, China) and stored at −20 °C. The ribosomal internal transcribed spacer (ITS), actin (ACT), glyceraldehyde-3-phosphate dehydrogenase (GAPDH), beta-tubulin (TUB2), partial sequences of the chitin synthase 1 (CHS-1), calmodulin (CAL), glutamine synthetase (GS), mating type protein, and the Apn2-Mat1-2 intergenic spacer (ApMat) were amplified. The protocols for amplification and the PCR primers used in this study are listed in Supplementary Table S2. Each 50 μL PCR mixture included 25 μL of Premix Taq^TM^ (Takara Biomedical Technology Company Limited, Beijing, China), 22 μL of ddH_2_O, 1 μL of each primer, and 1 μL of genomic DNA. PCR purification and sequencing were performed by ShangHai Huagene Biotech Company Limited, Shanghai, China.

### Phylogenetic analysis

The accession numbers of all sequences in this study were obtained from NCBI-GenBank and are listed in Supplementary Table S3. A phylogenetic tree was constructed using Multi-locus sequences. The dataset was assembled using MAFFT v. 7^43^ and manually adjusted using MEGA v. 6.0^44^. All gaps were treated as missing data. Nucleotide substitution models were generated using MrModeltest v. 2.3^45^, and the GTR + I + G model with gamma-distributed rate was selected for constructing all phylogenic trees. A maximum likelihood phylogenetic analysis of the dataset was performed with RAxML^46^. Markov Chain Monte Carlo (MCMC) sampling was used to reconstruct phylogenies in Mrbayes v. 3.2^47^. Analyses of 6 MCMC chains based on the full dataset were run for 1 × 10^7^ generations and sampled every 100 generations. The first 25% of the generations were discarded as burn-in. Figures of trees were created in FigTree v 1.3.1^48^.

### Morphological characterization

Mycelial discs (9 mm diameter) were taken from 5-day-old cultures, plated on PDA, and incubated at 25 °C in the dark. Daily growth rate was calculated after 5 days of growth and was based on values from three replicates. Colony characteristics were also recorded. Conidial, conidiophores, and appressoria characteristics were determined using methods described by Cai, *et al*.^4^. Additionally, appressoria were produced and measured using a slide culture technique and induced on synthetic nutrient-poor agar (SNA) medium. After 7 days, the shapes and sizes of 30 conidia, conidiophores, and appressoria were recorded (Eclipse 80i, Nikon, Japan).

### Pathogenicity tests

Inoculations were based on the method described by Liu, *et al*.^2^. Six strains were selected for pathogenicity testing: *C. aenigma* CGMCC 3.17883, *C. camelliae* CGMCC 3.17884, *C. endophytica* CGMCC 3.17887, *Colletotrichum* sp. CGMCC 3.17890, *C. siamense* CGMCC 3.17892, and *C. wuxiense* CGMCC 3.17894. Healthy and non-wounded mature tea leaves, collected from 5-year-old *Ca. sinensis* cv. *Longjing 43* grown in a tea garden in Hangzhou, Zhejiang province, China, were washed with tap water and then disinfected in 1% sodium hypochlorite for 3 min. Disinfected mature leaves were washed three times with sterilized water and then dried on the benchtop. Using sterile needles, 20 μL of conidial suspension (10^6^ spores/mL) was added to three wounded leaves for each strain. Leaves inoculated with sterile water were used as controls. The inoculated samples were laid on plastic petri dishes 12 cm in diameter and cultured in a growth cabinet at 25 °C with a light cycle of 12 h fluorescent light and 12 h darkness for 14 d. Finally, conidia of each strain were collected from diseased leaves and cultured on a new PDA plate. They were then checked for morphological characteristics to confirm Koch’s postulates^4^.

### Genealogical concordance phylogenetic species recognition analysis

We used the Genealogical Concordance Phylogenetic Species Recognition (GCPSR) model, as described by Liu, *et al*.^2^, by performing a pairwise homoplasy index (Φ_w_, PHI) test to analyze related but ambiguous species in the phylogenetic tree. The PHI test was performed in Splits Tree 4^49^,^50^ using 6-locus concatenated datasets (ITS, ACT, GAPDH, CAL, TUB2 and GS), and both the LogDet transformation and splits decomposition options were selected^51^. PHI results below a 0.05 threshold (Φ_w_ < 0.05) were considered indicative of significant recombination in the dataset.

### Prevalence of *Colletotrichum* species

The Isolation Rate (IR), calculated as IR% = (*Cx*/*Ct*) × 100, where *Cx* is the number of isolates of the same species and *Ct* was the total number of isolates^52^, was determined as a measure of the prevalence of *Colletotrichum* species in *Ca. sinensis* in China.

### Statistical Analysis

SPSS (SPSS Inc., USA) was used to conduct statistical analyses. The average value of all measurements including sizes of conidia, appressoria and daily growth rate were used for statistical analyses, and values were expressed as average ± standard error (av ± SE).

## Additional Information

**How to cite this article**: Wang, Y.-C. *et al*. Diverse *Colletotrichum* species cause anthracnose of tea plants (*Camellia sinensis* (L.) O. Kuntze) in China. *Sci. Rep.* **6**, 35287; doi: 10.1038/srep35287 (2016).

**Publisher’s note:** Springer Nature remains neutral with regard to jurisdictional claims in published maps and institutional affiliations.

## Supplementary Material

Supplementary Information

## Figures and Tables

**Table 1:** Geographical distribution of *Colletotrichum* species associated with *Ca. sinensis* in China.

Species	Collecting Location														
	AH	CQ	FJ	GD	GX	GZ	HN	HB	HuN	JS	JX	SX	SC	YN	ZJ
*C. acutatum*		⊥													
*C. aenigma*										*					
*C. alienum*											†				
*C. boninense*											†				
*C. camelliae*	*	*	*†	*		*†	*	*	*	*	*†	*	*	*	*
*C. cliviae*	*				†										*
*C. endophytica*														*	
*C. fioriniae*			*§								†		*		*
*C. fructicola*		*	*§	*	*†			*	*	*	†	*	*	*	*†
*C. gloeosporioides*	#										†				
*C. henanense*							†								
*C. jiangxiense*											†				
*C. karstii*			*§						*	*				*	*†
*Colletotrichum* sp.													*		
*C. siamense*			*§		*						*			*†	
*C. truncatum*															*
*C. wuxiense*										*					

AH: Anhui; CQ: Chongqiong; FJ: Fujian; GD: Guangdong; GX: Guangxi; GZ: Guizhou; HN: Guizhou; HB: Hubei; HuN: Hunan; JS: Jiangsu; JX: Jiangxi; SX: Shaanxi; SC: Sichuan; YN: Yunnan; ZJ: Zhejiang. This table includes data from Liu *et al*.^2^ (†), Guo *et al*.^3^ (#), Liu^28^ (§), Chen *et al*.^53^ (⊥), and our study (*).
