# Supplementary material for: Diverse Colletotrichum species cause anthracnose of tea plants (Camellia sinensis (L.) O. Kuntze) in China
Source: Sci Rep. 2016 Oct 26;6:35287. doi: 10.1038/srep35287 (PMC5080629; doi:10.1038/srep35287)
Supplement: Supplementary Information [file srep35287-s1.pdf]

Supplementary information

Diverse *Colletotrichum* species cause anthracnose of tea plants (*Camellia sinensis* (L.) O. Kuntze) in China

Yu-Chun Wang[1,2], Xin-Yuan Hao[1], Lu Wang[1], Bin Xiao[2], Xin-Chao Wang[1*] & Ya-Jun Yang[1,2*]

[1]Tea Research Institute, Chinese Academy of Agricultural Sciences/National Center for Tea Improvement/Key Laboratory of Tea Biology and Resources Utilization, Ministry of Agriculture, Hangzhou 310008, People's Republic of China

[2]College of Horticulture, Northwest A&F University, Yangling 712100, Shaanxi, People's Republic of China

*Correspondence to:
Xin-Chao Wang
Email: xcw75@tricaas.com

Ya-Jun Yang
Email: yjyang@tricaas.com

Supplementary Table S1. Collection data of *Colletotrichum* species from 15 provinces or cities in China

Species	Isolate NO.	Host	Location
C. aenigma	JS1A9	*Ca. sinensis*	Wuxi, Jiangsu
	JS1A29	*Ca. sinensis*	Wuxi, Jiangsu
C. camelliae	AH1B1	*Ca. sinensis* cv. *Keemenzhong*	Qimen, Anhui
	CQ1A10	*Ca. sinensis*	Yongchuan, Chongqing
	FJ1A5	*Ca. sinensis* cv. *Huangguanyin*	Fuzhou, Fujian
	GD1A4	*Ca. sinensis* cv. *Jinxuan*	Guangzhou, Guangdong
	GZ1A1	*Ca. sinensis* cv. *Fuding Dabaicha*	Tongren, Guizhou
	GZ1A2	*Ca. sinensis* cv. *Fuding Dabaicha*	Tongren, Guizhou
	GZ2A1	*Ca. sinensis* cv. *Qianmei 809*	Zunyi, Guizhou
	HB1A1	*Ca. sinensis*	Enshi, Hubei
	HB1A2	*Ca. sinensis*	Enshi, Hubei
	HEN1A2	*Ca. sinensis* cv. *Xinyang*	Xinyang, Henan
	HUN1A4	*Ca. sinensis* cv. *Baihaozao*	Changsha, Hunan
	JS1A3	*Ca. sinensis*	Wuxi, Jiangsu
	JS1A7	*Ca. sinensis*	Wuxi, Jiangsu
	JS1A10	*Ca. sinensis*	Wuxi, Jiangsu
	JS1A16	*Ca. sinensis*	Wuxi, Jiangsu
	JS1A17	*Ca. sinensis*	Wuxi, Jiangsu
	JS1A20	*Ca. sinensis*	Wuxi, Jiangsu
	JS1A31	*Ca. sinensis*	Wuxi, Jiangsu
	JS1A35	*Ca. sinensis*	Wuxi, Jiangsu
	JS1A41	*Ca. sinensis*	Wuxi, Jiangsu
	JS1A46	*Ca. sinensis*	Wuxi, Jiangsu
	JX1A2	*Ca. sinensis* cv. *Fuding Dabaicha*	Nanchang, Jiangxi
	SC2B7	*Ca. sinensis* cv. *Mingshan 131*	Meishan, Sichuan
	SH1A1	*Ca. sinensis* cv. *Ziyang*	Hanzhong, Shaanxi
	SH1B4	*Ca. sinensis* cv. *Fuding Dabaicha*	Hanzhong, Shaanxi
	SH1C1	*Ca. sinensis* cv. *Nanjiang 1*	Hanzhong, Shaanxi
	YN2A1	*Ca. sinensis*	Puer, Yunan
	YN2A2	*Ca. sinensis*	Puer, Yunan,
	ZJ1A5	*Ca. sinensis* cv. *Longjing 43*	Hangzhou, Zhejiang
	ZJ1A8	*Ca. sinensis* cv. *Longjing 43*	Hangzhou, Zhejiang
	ZJ1A11	*Ca. sinensis* cv. *Longjing 43*	Hangzhou, Zhejiang
	ZJ2A4	*Ca. sinensis*	Lishui, Zhejiang
C. cliviae	AH1A2	*Ca. sinensis* cv. *Keemenzhong*	Qimen, Anhui
	AH1B5	*Ca. sinensis* cv. *Keemenzhong*	Qimen, Anhui
	AH1B6	*Ca. sinensis* cv. *Keemenzhong*	Qimen, Anhui
	ZJ3A4	*Ca. sinensis* cv. *Longjing 43*	Hangzhou, Zhejiang
	ZJ3A9	*Ca. sinensis* cv. *Longjing 43*	Hangzhou, Zhejiang
C. endophytica	YN1A3	*Ca. sinensis* cv. *Menku*	Lincang, Yunnan
	YN1A4	*Ca. sinensis* cv. *Menku*	Lincang, Yunnan

	YN1A5	*Ca. sinensis* cv. *Menku*	Lincang, Yunnan
C. fioriniae	FJ1A1	*Ca. sinensis* cv. *Fuding Dahaocha*	Fuzhou, Fujian
	FJ1A2	*Ca. sinensis* cv. *Huangdan*	Fuzhou, Fujian
	SC3A1	*Ca. sinensis* cv. *Mingshanzao 132*	Meishan, Sichuan
	SC3A2	*Ca. sinensis* cv. *Mingshanzao 132*	Meishan, Sichuan
	ZJ1A1	*Ca. sinensis* cv. *Longjing 43*	Hangzhou, Zhejiang
	ZJ1A2	*Ca. sinensis* cv. *Longjing 43*	Hangzhou, Zhejiang
C. fructicola	CQ1A5	*Ca. sinensis*	Yongchuan, Chongqing
	FJ2B1	*Ca. sinensis* cv. *Benshan*	Fujian, Quanzhou
	FJ2C9	*Ca. sinensis* cv. *Maoxie*	Quanzhou, Fujian
	FJ2C12	*Ca. sinensis* cv. *Maoxie*	Quanzhou, Fujian
	FJ2D2	*Ca. sinensis* cv. *Tie guanyin*	Fujian, Quanzhou
	FJ2D3	*Ca. sinensis* cv. *Tie guanyin*	Fujian, Quanzhou
	GD1A5	*Ca. sinensis* cv. *Jinxuan*	Guangzhou, Guangdong
	GX1A1	*Ca. sinensis* cv. *Guilv 1*	Guilin, Guangxi
	GX2A4	*Ca. sinensis* cv. *Jiukang*	Guilin, Guangxi
	HB1A5	*Ca. sinensis*	Enshi, Hubei
	HUN2A1	*Ca. sinensis*	Changsha, Hunan
	JS1A6	*Ca. sinensis*	Wuxi, Jiangsu
	JS1A13	*Ca. sinensis*	Wuxi, Jiangsu
	JS1A21	*Ca. sinensis*	Wuxi, Jiangsu
	JS1A30	*Ca. sinensis*	Wuxi, Jiangsu
	JS1A37	*Ca. sinensis*	Wuxi, Jiangsu
	SC1A1	*Ca. sinensis* cv. *Zhongcha 302*	Yibin, Sichuan
	SC2A1	*Ca. sinensis* cv. *Anjibaicha*	Meishan, Sichuan
	SC2B2	*Ca. sinensis* cv. *Mingshan 131*	Meishan, Sichuan
	SH1A4	*Ca. sinensis* cv. *Ziyang*	Hanzhong, Shaanxi
	SH1B1	*Ca. sinensis* cv. *Fuding Dabaicha*	Hanzhong, Shaanxi
	YN1A1	*Ca. sinensis* cv. *Menku*	Lincang, Yunnan
	YN1A2	*Ca. sinensis* cv. *Menku*	Lincang, Yunnan
	YN2A10	*Ca. sinensis*	Puer, Yunan
	ZJ1A6	*Ca. sinensis* cv. *Longjing 43*	Hangzhou, Zhejiang
	ZJ1A7	*Ca. sinensis* cv. *Longjing 43*	Hangzhou, Zhejiang
	ZJ1A9	*Ca. sinensis* cv. *Longjing 43*	Hangzhou, Zhejiang
	ZJ1A10	*Ca. sinensis* cv. *Longjing 43*	Hangzhou, Zhejiang
	ZJ1B1	*Ca. sinensis* cv. *Zhongcha 108*	Hangzhou, Zhejiang
	ZJ1B2	*Ca. sinensis* cv. *Zhongcha 108*	Hangzhou, Zhejiang
	ZJ3A6	*Ca. sinensis* cv. *Longjing 43*	Hangzhou, Zhejiang
	ZJ3A8	*Ca. sinensis* cv. *Longjing 43*	Hangzhou, Zhejiang
	ZJ3A10	*Ca. sinensis* cv. *Longjing 43*	Hangzhou, Zhejiang
C. karstii	FJ2A1	*Ca. sinensis* cv. *Huangjingui*	Quanzhou, Fujian
	FJ2A2	*Ca. sinensis* cv. *Huangjingui*	Quanzhou, Fujian
	FJ2C1	*Ca. sinensis* cv. *Maoxie*	Quanzhou, Fujian
	FJ2C6	*Ca. sinensis* cv. *Maoxie*	Quanzhou, Fujian

	FJ2C8	*Ca. sinensis* cv. *Maoxie*	Quanzhou, Fujian
	FJ2C11	*Ca. sinensis* cv. *Maoxie*	Quanzhou, Fujian
	FJ2C13	*Ca. sinensis* cv. *Maoxie*	Quanzhou, Fujian
	FJ2D1	*Ca. sinensis* cv. *Tie guanyin*	Quanzhou, Fujian
	HUN2A7	*Ca. sinensis*	Changsha, Hunan
	JS1A8	*Ca. sinensis*	Wuxi, Jiangsu
	JS1A14	*Ca. sinensis*	Wuxi, Jiangsu
	YN1A6	*Ca. sinensis* cv. *Menku*	Lincang, Yunnan
	ZJ3A1	*Ca. sinensis* cv. *Longjing43*	Hangzhou, Zhejiang
Colletotrichum sp.	SC3A3	*Ca. sinensis* cv. *Mingshanzao 132*	Meishan, Sichuan
C. siamense	FJ1A3	*Ca. sinensis* cv. *Fujian Shuixian*	Fuzhou, Fujian
	FJ1A4	*Ca. sinensis* cv. *Fuding Dabaicha*	Fuzhou, Fujian
	FJ2D4	*Ca. sinensis* cv. *Tie guanyin*	Fujian, Quanzhou
	GX2A1	*Ca. sinensis* cv. *Jiukang*	Guilin, Guangxi
	GX2A3	*Ca. sinensis* cv. *Jiukang*	Guilin, Guangxi
	JX1A1	*Ca. sinensis* cv. *Fuding Dabaicha*	Nanchang, Jiangxi
	JX1A3	*Ca. sinensis* cv. *Fuding Dabaicha*	Nanchang, Jiangxi
	YN2A9	*Ca. sinensis*	Puer, Yunan
C. truncatum	ZJ3A3	*Ca. sinensis* cv. *Longjing43*	Hangzhou, Zhejiang
C. wuxiense	JS1A32	*Ca. sinensis*	Wuxi, Jiangsu
	JS1A44	*Ca. sinensis*	Wuxi, Jiangsu

Supplementary Table S2. Primers used in this study

Gene	Product name	Primer	Sequence	Reference
ACT	Actin	ACT-512F	ATG TGC AAG GCC GGT TTC GC	Carbone and Kohn[1]
		ACT-783R	TAC GAG TCC TTC TGG CCC AT	Carbone and Kohn[1]
ApMat	Mating type protein and Apn2-Mat1-2 intergenic spacer	AMF1	TCA TTC TAC GTA TGT GCC CG	Silva *et al.*[2]
		AMR1	CCA GAA ATA CAC CGA ACT TGC	Silva *et al.*[2]
CAL	Calmodulin	CL1C	GAA TTC AAG GAG GCC TTC TC	Weir *et al.*[3]
		CL2C	CTT CTG CAT CAT GAG CTG GAC	Weir *et al.*[3]
CHS-1	Chitin synthase	CHS-79F	TGG GGC AAG GAT GCT TGG AAG AAG	Carbone and Kohn[1]
		CHS-345R	TGG AAG AAC CAT CTG TGA GAG TTG	Carbone and Kohn[1]
GAPDH	Glyceraldehyde-3-phosphate dehydrogenase	GDF	GCC GTC AAC GAC CCC TTC ATT GA	Templeton *et al.*[4]
		GDR	GGG TGG AGT CGT ACT TGA GCA TGT	Templeton *et al.*[4]
GS	Glutamine synthetase	GSF1	ATG GCC GAG TAC ATC TGG	Stephenson *et al.*[5]
		GSR1	GCC GGT GGA GGA ACC GTC G	Stephenson *et al.*[5]
ITS	Internal transcribed spacer	ITS-1F	CTT GGT CAT TTA GAG GAA GTA A	Gardes and Bruns[6]
		ITS-4	TCC TCC GCT TAT TGA TAT GC	White *et al.*[7]
TUB2	β-Tubulin 2	T1	AAC ATG CGT GAG ATT GTA AGT	O'Donnell and Cigelnik[8]
		Bt2b	ACC CTC AGT GTA GTG ACC CTT GGC	Glass and Donaldson[9]

4 **Supplementary Table S3.** Isolates of the *Colletotrichum* species studied and GenBank accession numbers of the generated sequences

Species	Isolate /Culture collection	GenBank accessions							
		ITS	ACT	GAPDH	CAL	CHS-1	TUB2	GS	ApMat
C. acutatum	CBS 112996, ATCC 56816*	JQ005776	JQ005839	JQ948677		JQ005797	JQ005860		
C. aenigma	ICMP 18608*	JX010244	JX009443	JX010044	JX009683	JX009774	JX010389	JX010078	KM360143
	**JS1A9**	**KU251549**	**KU251630**	**KU252003**	**KU251793**	**KU251897**	**KU252156**	**KU252080**	
	**JS1A29, CGMCC 3.17883**	**KU251548**	**KU251629**	**KU252002**	**KU251792**	**KU251896**	**KU252155**	**KU252079**	
C.aeschynomenes	ICMP 17673, ATCC 201874*	JX010176	JX009483	JX009930	JX009721	JX009799	JX010392	JX010081	KM360145
C. alatae	CBS 304.67, ICMP 17919*	JX010190	JX009471	JX009990	JX009738	JX009837	JX010383	JX010065	KC888932
C. alienum	ICMP 12071*	JX010251	JX009572	JX010028	JX009654	JX009882	JX010411	JX010101	KM360144
C. aotearoa	ICMP 18537*	JX010205	JX009564	JX010005	JX009611	JX009853	JX010420	JX010113	KC888930
	ICMP 18532	JX010220	JX009544	JX009906	JX009614	JX009764	JX010421	JX010108	
C. asianum	ICMP 18580, CBS 130418*	FJ972612	FJ907424	FJ972576	FJ917506	JX009867	FJ907439	JX010096	FR718814
C. boninense	CBS 123755, MAFF 305972*	JQ005153	JQ005501	JQ005240	JQ005674	JQ005327	JQ005588		
C. brasiliense	CBS 128501*	JQ005235	JQ005583	JQ005322		JQ005409	JQ005669		
C. brisbanense	CBS 292.67*	JQ948291	JQ949612	JQ948621		JQ948952	JQ949942		
C. camelliae	CGMCC 3.14925, LC1364*	KJ955081	KJ954363	KJ954782	KJ954634		KJ955230	KJ954932	KJ954497
	LC3270, LF492	KJ955151	KJ954428	KJ954852	KJ954703		KJ955298	KJ955002	KJ954563
	ICMP 10643, LF897	JX010224	JX009540	JX009908	JX009630	JX009891	JX010436	JX010119	KJ954625

	ICMP 18542, LF899	JX010223	JX009488	JX009994	JX009628	JX009857	JX010429	JX010118	KJ954627
	ICMP 10646, LF898	JX010225	JX009563	JX009993	JX009629	JX009892	JX010437	JX010117	KJ954626
	**AH1B1**	**KU251562**	**KU251643**	**KU252016**	**KU251806**	**KU251910**	**KU252170**	**KU252083**	**KU251704**
	**CQ1A10**	**KU251575**	**KU251656**	**KU252029**	**KU251817**	**KU251923**	**KU252181**	**KU252065**	**KU251705**
	**FJ1A5**	**KU251576**	**KU251657**	**KU252030**	**KU251818**	**KU251924**	**KU252182**		
	**GD1A4**	**KU251563**	**KU251644**	**KU252017**	**KU251807**	**KU251911**	**KU252171**	**KU252084**	**KU251711**
	**GZ1A1**	**KU251564**	**KU251645**	**KU252018**	**KU251808**	**KU251912**	**KU252172**	**KU252085**	**KU251715**
	**GZ1A2**	**KU251577**	**KU251658**	**KU252031**	**KU251819**	**KU251925**	**KU252183**		
	**GZ2A1**	**KU251578**	**KU251659**	**KU252032**	**KU251820**	**KU251926**	**KU252184**		
	**HB1A1**	**KU251579**	**KU251660**	**KU252033**	**KU251821**	**KU251927**	**KU252185**		
	**HB1A2**	**KU251567**	**KU251648**	**KU252021**	**KU251810**	**KU251915**	**KU252198**	**KU252087**	**KU251716**
	**HEN1A2**	**KU251580**	**KU251661**	**KU252034**	**KU251822**	**KU251928**	**KU252186**		
	**HUN1A4**	**KU251565**	**KU251646**	**KU252019**	**KU251838**	**KU251913**	**KU252173**	**KU252086**	**KU251718**
	**JS1A3**	**KU251583**	**KU251664**	**KU252037**	**KU251825**	**KU251931**	**KU252189**		
	**JS1A7**	**KU251586**	**KU251667**	**KU252040**	**KU251828**	**KU251934**	**KU252192**		
	**JS1A10**	**KU251572**	**KU251653**	**KU252026**	**KU251840**	**KU251920**	**KU252178**	**KU252092**	**KU251719**
	**JS1A16**	**KU251581**	**KU251662**	**KU252035**	**KU251823**	**KU251929**	**KU252187**		
	**JS1A17**	**KU251574**	**KU251655**	**KU252028**	**KU251816**	**KU251922**	**KU252180**	**KU252093**	**KU251720**
	**JS1A20**	**KU251582**	**KU251663**	**KU252036**	**KU251824**	**KU251930**	**KU252188**		
	**JS1A31**	**KU251584**	**KU251665**	**KU252038**	**KU251826**	**KU251932**	**KU252190**		
	**JS1A35, CGMCC 3.17884**	**KU251595**	**KU251676**	**KU252049**	**KU251835**	**KU251943**	**KU252157**	**KU252089**	**KU251723**
	**JS1A41**	**KU251568**	**KU251649**	**KU252022**	**KU251811**	**KU251916**	**KU252199**	**KU252094**	**KU251725**
	**JS1A46**	**KU251585**	**KU251666**	**KU252039**	**KU251827**	**KU251933**	**KU252191**		
	**JX1A2**	**KU251587**	**KU251668**	**KU252041**	**KU251829**	**KU251935**	**KU252193**		
	**SC2B7**	**KU251588**	**KU251669**	**KU252042**	**KU251830**	**KU251936**	**KU252194**		

	**SH1A1**	**KU251594**	**KU251675**	**KU252048**	**KU251791**	**KU251942**	**KU252197**	**KU252088**	
	**SH1B4**	**KU251596**	**KU251677**	**KU252050**	**KU251836**	**KU251944**	**KU252158**	**KU252098**	**KU251731**
	**SH1C1**	**KU251589**	**KU251670**	**KU252043**	**KU251831**	**KU251937**	**KU252195**		
	**YN2A1**	**KU251597**	**KU251678**	**KU252051**	**KU251837**	**KU251945**	**KU252151**	**KU252091**	**KU251736**
	**YN2A2**	**KU251566**	**KU251647**	**KU252020**	**KU251809**	**KU251914**	**KU252174**	**KU252090**	**KU251737**
	**ZJ1A5**	**KU251571**	**KU251652**	**KU252025**	**KU251814**	**KU251919**	**KU252177**	**KU252099**	**KU251741**
	**ZJ1A8**	**KU251590**	**KU251671**	**KU252044**	**KU251832**	**KU251938**	**KU252196**		
	**ZJ1A11**	**KU251570**	**KU251651**	**KU252024**	**KU251813**	**KU251918**	**KU252176**	**KU252096**	**KU251740**
	**ZJ2A4**	**KU251573**	**KU251654**	**KU252027**	**KU251815**	**KU251921**	**KU252179**	**KU252097**	**KU251745**
C. clidemiae	ICMP 18658*	JX010265	JX009537	JX009989	JX009645	JX009877	JX010438	JX010129	KC888929
	ICMP 18706	JX010274	JX009476	JX009909	JX009639	JX009777	JX010439	JX010128	
C. cordylinicola	MFLUCC 090551, ICMP 18579*	JX010226	HM470235	JX009975	HM470238	JX009864	JX010440	JX010122	JQ899274
C. cliviae	CBS 125375*	GQ485607	GQ856777	GQ856756		GQ856722	GQ849440		
	LC3546, CGMCC 3.17358	KJ955215	KJ954483	KJ954916			KJ955361		
	**AH1A2**	**KU251492**	**KU251679**	**KU251946**		**KU251841**	**KU252103**		
	**AH1B5, CGMCC 3.17885**	**KU251493**	**KU251680**	**KU251947**		**KU251842**	**KU252105**		
	**AH1B6**	**KU251494**	**KU251681**	**KU251948**		**KU251843**	**KU252106**		
	**ZJ3A4**	**KU251515**	**KU251702**	**KU251969**		**KU251864**	**KU252107**		
	**ZJ3A9**	**KU251516**	**KU251703**	**KU251970**		**KU251865**	**KU252104**		
C. coccodes	CBS 369.75*	HM171679	HM171667	HM171673		JX546681	JX546873		
C. colombiense	CBS 129818*	JQ005174	JQ005522	JQ005261		JQ005348	JQ005608		
C. constrictum	CBS 128504*	JQ005238	JQ005586	JQ005325		JQ005412	JQ005672		
C. curcumae	IMI 288937*	GU227893	GU227991	GU228285		GU228383	GU228187		

C. dacrycarpi	CBS 130241*	JQ005236	JQ005584	JQ005323		JQ005410	JQ005670		
C. dianesei	CMM4083, MFLU 1300058*	KC329779	KC517298	KC517194	KC517209		KC517254	KC430894	
C. endophytica	MFLUCC 130418, LC0324*	KC633854	KF306258	KC832854			KC810018		
	MFLUCC 10-0676	KF242123	KF157827	KF242181	KF254846		KF254857	KF242154	
	**YN1A3, CGMCC 3.17886**	**KU251559**	**KU251640**	**KU252013**	**KU251802**	**KU251907**	**KU252167**	**KU252076**	**KU251733**
	**YN1A4, CGMCC 3.17887**	**KU251561**	**KU251642**	**KU252015**	**KU251804**	**KU251909**	**KU252169**	**KU252078**	**KU251734**
	**YN1A5**	**KU251560**	**KU251641**	**KU252014**	**KU251803**	**KU251908**	**KU252168**	**KU252077**	**KU251735**
C. fioriniae	CBS 127600	JQ948308	JQ949629	JQ948638		JQ948969	JQ949959		
	CBS 128517*	EF464594	JQ949613	EF593344		JQ948953	EF593325		
	CBS 129916	JQ948317	JQ949638	JQ948647		JQ948978	JQ949968		
	CBS 129948	JQ948344	JQ949665	JQ948674		JQ949005	JQ949995		
	CBS 200.35	JQ948293	JQ949614	JQ948623		JQ948954	JQ949944		
	**FJ1A1**	**KU251495**	**KU251682**	**KU251949**		**KU251844**	**KU252203**		
	**FJ1A2**	**KU251496**	**KU251683**	**KU251950**		**KU251845**	**KU252204**		
	**SC3A1**	**KU251508**	**KU251695**	**KU251962**		**KU251857**	**KU252205**		
	**SC3A2, CGMCC 3.17888**	**KU251509**	**KU251696**	**KU251963**		**KU251858**	**KU252206**		
	**ZJ1A1**	**KU251511**	**KU251698**	**KU251965**		**KU251860**	**KU252207**		
	**ZJ1A2**	**KU251512**	**KU251699**	**KU251966**		**KU251861**	**KU252208**		
C. fructicola	CBS 125397, ICMP 18646	JX010173	JX009581	JX010032	JX009674	JX009874	JX010409	JX010099	
	ICMP 18581, CBS	JX010165	FJ907426	JX010033	FJ917508	JX009866	JX010405	JX010095	JQ807838

130416*								
LC3427, LF649	KJ955191	KJ954464	KJ954892	KJ954742		KJ955338	KJ955041	KJ954597
LC3666, LF896, ICMP 18656	KJ955221	KJ954489	KJ954922	KJ954772		KJ955366	KJ955071	KJ954624
**CQ1A5, CGMCC 3.17889**	**KU251520**	**KU251601**	**KU251974**	**KU251763**	**KU251868**	**KU252152**	**KU252064**	**KU251706**
**FJ2B1**	**KU251529**	**KU251610**	**KU251983**	**KU251772**	**KU251877**	**KU252132**		
**FJ2C9**	**KU251530**	**KU251611**	**KU251984**	**KU251773**	**KU251878**	**KU252133**		
**FJ2C12**	**KU251540**	**KU251621**	**KU251994**	**KU251783**	**KU251888**	**KU252143**	**KU252053**	**KU251709**
**FJ2D2**	**KU251531**	**KU251612**	**KU251985**	**KU251774**	**KU251879**	**KU252134**		
**FJ2D3**	**KU251547**	**KU251628**	**KU252001**	**KU251790**	**KU251895**	**KU252149**	**KU252068**	
**GD1A5**	**KU251532**	**KU251613**	**KU251986**	**KU251775**	**KU251880**	**KU252135**		
**GX1A1**	**KU251533**	**KU251614**	**KU251987**	**KU251776**	**KU251881**	**KU252136**		
**GX2A4**	**KU251543**	**KU251624**	**KU251997**	**KU251786**	**KU251891**	**KU252153**	**KU252056**	**KU251714**
**HB1A5**	**KU251523**	**KU251604**	**KU251977**	**KU251766**	**KU251871**	**KU252126**	**KU252082**	**KU251717**
**HUN2A1**	**KU251524**	**KU251605**	**KU251978**	**KU251767**	**KU251872**	**KU252127**		
**JS1A6**	**KU251534**	**KU251615**	**KU251988**	**KU251777**	**KU251882**	**KU252137**		
**JS1A13**	**KU251527**	**KU251608**	**KU251981**	**KU251770**	**KU251875**	**KU252130**		
**JS1A21**	**KU251525**	**KU251606**	**KU251979**	**KU251768**	**KU251873**	**KU252128**		
**JS1A30**	**KU251544**	**KU251625**	**KU251998**	**KU251787**	**KU251892**	**KU252146**	**KU252058**	**KU251721**
**JS1A37**	**KU251521**	**KU251602**	**KU251975**	**KU251764**	**KU251869**	**KU252154**	**KU252061**	**KU251724**
**SC1A1**	**KU251541**	**KU251622**	**KU251995**	**KU251784**	**KU251889**	**KU252144**	**KU252054**	**KU251726**
**SC2A1**	**KU251528**	**KU251609**	**KU251982**	**KU251771**	**KU251876**	**KU252131**		**KU251729**
**SC2B2**	**KU251526**	**KU251607**	**KU251980**	**KU251769**	**KU251874**	**KU252129**		
**SH1A4**	**KU251550**	**KU251631**	**KU252004**	**KU251805**	**KU251898**	**KU252150**	**KU252063**	
**SH1B1**	**KU251569**	**KU251650**	**KU252023**	**KU251812**	**KU251917**	**KU252175**	**KU252095**	

	**YN1A1**	**KU251539**	**KU251620**	**KU251993**	**KU251782**	**KU251887**	**KU252142**	**KU252066**	**KU251732**
	**YN1A2**	**KU251537**	**KU251618**	**KU251991**	**KU251780**	**KU251885**	**KU252140**		
	**YN2A10**	**KU251538**	**KU251619**	**KU251992**	**KU251781**	**KU251886**	**KU252141**		
	**ZJ1A6**	**KU251535**	**KU251616**	**KU251989**	**KU251778**	**KU251883**	**KU252138**		
	**ZJ1A7**	**KU251545**	**KU251626**	**KU251999**	**KU251788**	**KU251893**	**KU252147**	**KU252057**	**KU251742**
	**ZJ1A9**	**KU251546**	**KU251627**	**KU252000**	**KU251789**	**KU251894**	**KU252148**	**KU252067**	**KU251743**
	**ZJ1A10**	**KU251522**	**KU251603**	**KU251976**	**KU251765**	**KU251870**	**KU252125**	**KU252062**	**KU251739**
	**ZJ1B1**	**KU251536**	**KU251617**	**KU251990**	**KU251779**	**KU251884**	**KU252139**		
	**ZJ1B2**	**KU251542**	**KU251623**	**KU251996**	**KU251785**	**KU251890**	**KU252145**	**KU252055**	**KU251744**
	**ZJ3A6**	**KU251519**	**KU251600**	**KU251973**	**KU251762**	**KU251867**	**KU252124**	**KU252060**	**KU251746**
	**ZJ3A8**	**KU251518**	**KU251599**	**KU251972**	**KU251761**	**KU251866**	**KU252123**	**KU252059**	**KU251747**
	**ZJ3A10**	**KU251517**	**KU251598**	**KU251971**	**KU251760**		**KU252122**	**KU252052**	
C. fructivorum	Coll1092, BPI 884114, CBS 133135	JX145133					JX145184		
	Coll1414, BPI 884103, CBS 133125*	JX145145					JX145196		
C. gloeosporioides	IMI 356878, ICMP 17821, CBS 112999*	JQ005152	JQ005500	JQ005239	JQ005673	JQ005326	JX010445	JX010085	JQ807843
C. godetiae	CBS 133.44*	JQ948402	JQ949723	JQ948733		JQ949063	JQ950053		
C. grevilleae	CBS 132879*	KC297078	KC296941	KC297010	KC296963	KC296987	KC297102	KC297033	
C. hebeiense	CGMCC3.17464*	KF156863	KF377532	KF377495		KF289008	KF288975		
C. henanense	LC3030, CGMCC 3.17354, LF238*	KJ955109	KM023257	KJ954810	KJ954662		KJ955257	KJ954960	KJ954524
C. horii	NBRC 7478, ICMP 10492, MTCC 10841*	GQ329690	JX009438	GQ329681	JX009604	JX009752	JX010450	JX010137	JQ807840
	ICMP 12942	GQ329687	JX009533	GQ329685	JX009603	JX009748	JX010375	JX010072	

C. jiangxiense	LC3460, CGMCC 3.17362, LF684	KJ955198	KJ954469	KJ954899	KJ954749		KJ955345	KJ955048	KJ954604
	LC3463, CGMCC 3.17363, LF687*	KJ955201	KJ954471	KJ954902	KJ954752		KJ955348	KJ955051	KJ954607
C. johnstonii	CBS 128532, ICMP 12926*	JQ948444	JQ949765	JQ948775		JQ949105	JQ950095		
C. karstii	CBS 118401	JQ005192	JQ005540	JQ005279		JQ005366	JQ005626		
	CBS 128524, ICMP 18588	JQ005195	JQ005543	JQ005282		JQ005369	JQ005629		
	CBS 129833	JQ005175	JQ005523	JQ005262		JQ005349	JQ005609		
	CBS 132134, CGMCC 3.14194*	HM585409	HM581995	HM585391		HM582023	HM585428		
	MAFF 306204, ICMP 18597	JQ005196	JQ005544	JQ005283		JQ005370	JQ005630		
	**FJ2A1**	**KU251497**	**KU251684**	**KU251951**	**KU251748**	**KU251846**	**KU252108**		
	**FJ2A2**	**KU251498**	**KU251685**	**KU251952**	**KU251749**	**KU251847**	**KU252109**		
	**FJ2C1**	**KU251499**	**KU251686**	**KU251953**	**KU251750**	**KU251848**	**KU252111**		
	**FJ2C6**	**KU251502**	**KU251689**	**KU251956**	**KU251753**	**KU251851**	**KU252114**		
	**FJ2C8**	**KU251503**	**KU251690**	**KU251957**	**KU251754**	**KU251852**	**KU252115**		
	**FJ2C11**	**KU251500**	**KU251687**	**KU251954**	**KU251751**	**KU251849**	**KU252112**		
	**FJ2C13**	**KU251501**	**KU251688**	**KU251955**	**KU251752**	**KU251850**	**KU252113**		
	**FJ2D1**	**KU251504**	**KU251691**	**KU251958**	**KU251755**	**KU251853**	**KU252116**		
	**HUN2A7**	**KU251505**	**KU251692**	**KU251959**	**KU251756**	**KU251854**	**KU252117**		
	**JS1A8**	**KU251507**	**KU251694**	**KU251961**	**KU251758**	**KU251856**	**KU252119**		
	**JS1A14**	**KU251506**	**KU251693**	**KU251960**	**KU251757**	**KU251855**	**KU252118**		
	**YN1A6**	**KU251510**	**KU251697**	**KU251964**	**KU251759**	**KU251859**	**KU252110**		

	**ZJ3A1**	**KU251513**	**KU251700**	**KU251967**		**KU251862**	**KU252120**		
C. kahawae subsp. *ciggaro*	ICMP 12952	JX010214	JX009431	JX009971	JX009648	JX009757	JX010426	JX010126	
	IMI 359911, ICMP 17931,CBS 12988	JX010236	JX009475	JX009965	JX009637	JX009832	JX010428	JX010121	
	ICMP 18534	JX010227	JX009473	JX009904	JX009634	JX009765	JX010427	JX010116	HE655657
	ICMP 18539*	JX010230	JX009523	JX009966	JX009635	JX009800	JX010434	JX010132	
C. kahawae subsp. *kahawae*	IMI 301220, ICMP 17811	JX010233	JX009555	JX009970	JX009641	JX009817	JX010430	JX010131	
	IMI 319418, ICMP 17816*	JX010231	JX009452	JX010012	JX009642	JX009813	JX010444	JX010130	JQ894579
	IMI 361501, ICMP 17905	JX010232	JX009561	JX010046	JX009644	JX009816	JX010431	JX010127	
C. lindemuthianum	CBS 144.31*	JQ005779	JQ005842	JX546712		JQ005800	JQ005863		
C. melanocaulon	Coll131, BPI 884113, CBS 133251*	JX145144					JX145195		JX145313
C. musae	CBS 116870, ICMP 19119, MTCC 11349*	JX010146	JX009433	JX010050	JX009742	JX009896	HQ596280	JX010103	KC888926
	IMI 52264, ICMP 17817	JX010142	JX009432	JX010015	JX009689	JX009815	JX010395	JX010084	
C. nupharicola	CBS 470.96, ICMP 18187*	JX010187	JX009437	JX009972	JX009663	JX009835	JX010398	JX010088	JX145319
	CBS 472.96, ICMP 17940	JX010188	JX009582	JX010031	JX009662	JX009836	JX010399	JX010089	
C. orchidearum	CBS 632.80*	JQ948151	JQ949472	JQ948481		JQ948812	JQ949802		
C. phormii	CBS 118194*	JQ948446	JQ949767	JQ948777		JQ949107	JQ950097		

C. phyllanthi	CBS 175.67*	JQ005221	JQ005569	JQ005308		JQ005395	JQ005655		
C. proteae	CBS 132882*	KC297079	KC296940	KC297009	KC296960	KC296986	KC297101	KC297032	
C. psidii	CBS 145.29, ICMP 19120*	JX010219	JX009515	JX009967	JX009743	JX009901	JX010443	JX010133	KC888931
C. queenslandicum	ICMP 1778*	JX010276	JX009447	JX009934	JX009691	JX009899	JX010414	JX010104	KC888928
C. rhexiae	Coll1026, BPI 884112, CBS 133134*	JX145128					JX145179		JX145290
	Coll877, BPI 884110, CBS 133132	JX145157					JX145209		JX145302
C. rusci	CBS 119206*	GU227818	GU227916	GU228210		GU228308	GU228112		
C. salicis	CBS 607.94*	JQ948460	JQ949781	JQ948791		JQ949121	JQ950111		
C. salsolae	ICMP 19051*	JX010242	JX009562	JX009916	JX009696	JX009863	JX010403	JX010093	KC888925
Colletotrichum sp.	**SC3A3, CGMCC 3.17890**	**KU251593**	**KU251674**	**KU252047**	**KU251839**	**KU251941**	**KU252202**	**KU252100**	**KU251730**
C. siamense	CBS 125378, ICMP 18642	JX010278	GQ856775	JX010019	JX009709	GQ856730	JX010410	JX010100	JQ899283
	CBS 130420, ICMP 19118	HM131511	HM131507	HM131497	JX009713	JX009895	JX010415	JX010105	JQ807841
	CMM3814	KC702994	KC702922	KC702955	KC992372	KC598113	KM404170		KJ155453
	GC01	KC790972	KC790619	KC790733	KF451951	KF451986	KC790866		KC790671
	GZAAS 5.09506*	JQ247633	JQ247657	JQ247609	JQ247596		JQ247644	JQ247621	
	ICMP 18578, CBS 130417*	JX010171	FJ907423	JX009924	FJ917505	JX009865	JX010404	JX010094	JQ899289
	ITCC 6066	JN390914	KC790651	KC790764	KF451966	KF452001	KC790897		KC790701
	ITCC 6166	JN390871	KC790662	KC790773			KC790908		KC790712
	LC2974, LF182	KJ955093	KJ954375	KJ954794	KJ954646		KJ955242	KJ954944	KJ954509

	MTCC 11599	JQ894681	JQ894546	JQ894632	KC790791	JQ894617	JQ894602		JQ894582
	**FJ1A3**	**KU251551**	**KU251632**	**KU252005**	**KU251794**	**KU251899**	**KU252159**	**KU252069**	**KU251707**
	**FJ1A4**	**KU251553**	**KU251634**	**KU252007**	**KU251796**	**KU251901**	**KU252160**	**KU252071**	**KU251708**
	**FJ2D4**	**KU251552**	**KU251633**	**KU252006**	**KU251795**	**KU251900**	**KU252161**	**KU252070**	**KU251710**
	**GX2A1, CGMCC 3.17891**	**KU251555**	**KU251636**	**KU252009**	**KU251798**	**KU251903**	**KU252163**	**KU252073**	**KU251712**
	**GX2A3, CGMCC 3.17892**	**KU251554**	**KU251635**	**KU252008**	**KU251797**	**KU251902**	**KU252162**	**KU252072**	**KU251713**
	**JX1A1**	**KU251556**	**KU251637**	**KU252010**	**KU251799**	**KU251904**	**KU252164**	**KU252074**	**KU251727**
	**JX1A3**	**KU251557**	**KU251638**	**KU252011**	**KU251800**	**KU251905**	**KU252165**	**KU252075**	**KU251728**
	**YN2A9**	**KU251558**	**KU251639**	**KU252012**	**KU251801**	**KU251906**	**KU252166**	**KU252081**	**KU251738**
C. syzygicola	MFLUCC 10-0624[*]	KF242094	KF157801	KF242156	KF254859		KF254880	KF242125	
C. temperatum	Coll1103, BPI 884098, CBS 133120	JX145135					JX145186		JX145297
	Coll883, BPI 884100, CBS 133122[*]	JX145159					JX145211		JX145298
C. theobromicola	CBS 142.31, ICMP 17927, MTCC 10325	JX010286	JX009516	JX010024	JX009592	JX009830	JX010373	JX010064	JQ807844
	MTCC 11350, CBS 124945, ICMP 18649[*]	JX010294	JX009444	JX010006	JX009591	JX009869	JX010447	JX010139	KC790726
C. ti	ICMP 4832[*]	JX010269	JX009520	JX009952	JX009649	JX009898	JX010442	JX010123	KM360146
C. tropicale	CBS 124949, ICMP 18653, MTCC 11371[*]	JX010264	JX009489	JX010007	JX009719	JX009870	JX010407	JX010097	KC790728
C. truncatum	CBS 151.35[*]	GU227862	GU227960	GU228254		GU228352	GU228156		
	CBS 182.52	GU227866	GU227964	GU228258		GU228356	GU228160		
	CBS 195.32	GU227865	GU227963	GU228257		GU228355	GU228159		

	CBS 667.88	GU227891	GU227989	GU228283		GU228381	GU228185		
	**ZJ3A3, CGMCC 3.17893**	**KU251514**	**KU251701**	**KU251968**		**KU251863**	**KU252121**		
C. viniferum	GZAAS 5.08601*	JN412804	JN412795	JN412798	JQ309639		JN412813	JN412787	
C. walleri	CBS 125472*	JQ948275	JQ949596	JQ948605		JQ948936	JQ949926		
C. wuxiense	**JS1A32, CGMCC 3.17894**	**KU251591**	**KU251672**	**KU252045**	**KU251833**	**KU251939**	**KU252200**	**KU252101**	**KU251722**
	**JS1A44**	**KU251592**	**KU251673**	**KU252046**	**KU251834**	**KU251940**	**KU252201**	**KU252102**	
C. xanthorrhoeae	BRIP 45094, ICMP 17903, CBS 127831*	JX010261	JX009478	JX009927	JX009653	JX009823	JX010448	JX010138	KC790689

CGMCC: China General Microbiological Culture Collection; ATCC: American Type Culture Collection; BPI: U.S. National Fungus Collections, USA; BRIP: Plant Pathology Herbarium, Department of Employment, Economic, Development and Innovation, Queensland, Australia; CBS: Culture collection of the Centraalbureau voor Schimmelcultures, Fungal Biodiversity Centre, Utrecht, The Netherlands; GZAAS: Guizhou Academy of Agricultural Sciences Herbarium, China; ICMP: International Collection of Microorganisms from Plants, Auckland, New Zealand; IMI: Culture collection of CABI Europe UK Centre, Egham, UK; ITCC: Indian Type Culture Collection, New Delhi, India; LC: Working collection of Lei Cai, housed at CAS, China; LF: Working collection of Fang Liu, housed at CAS, China; MAFF: MAFF Genebank Project, Ministry of Agriculture, Forestry and Fisheries, Tsukuba, Japan; MFLUCC: Mae Fah Luang University Culture Collection, Chiang Rai, Thailand; MTCC: Microbial type culture collection and gene bank, India; NBRC: NITE Biological Resource Centre, Japan.

* = ex-type culture. Strains studied in this paper are in bold.

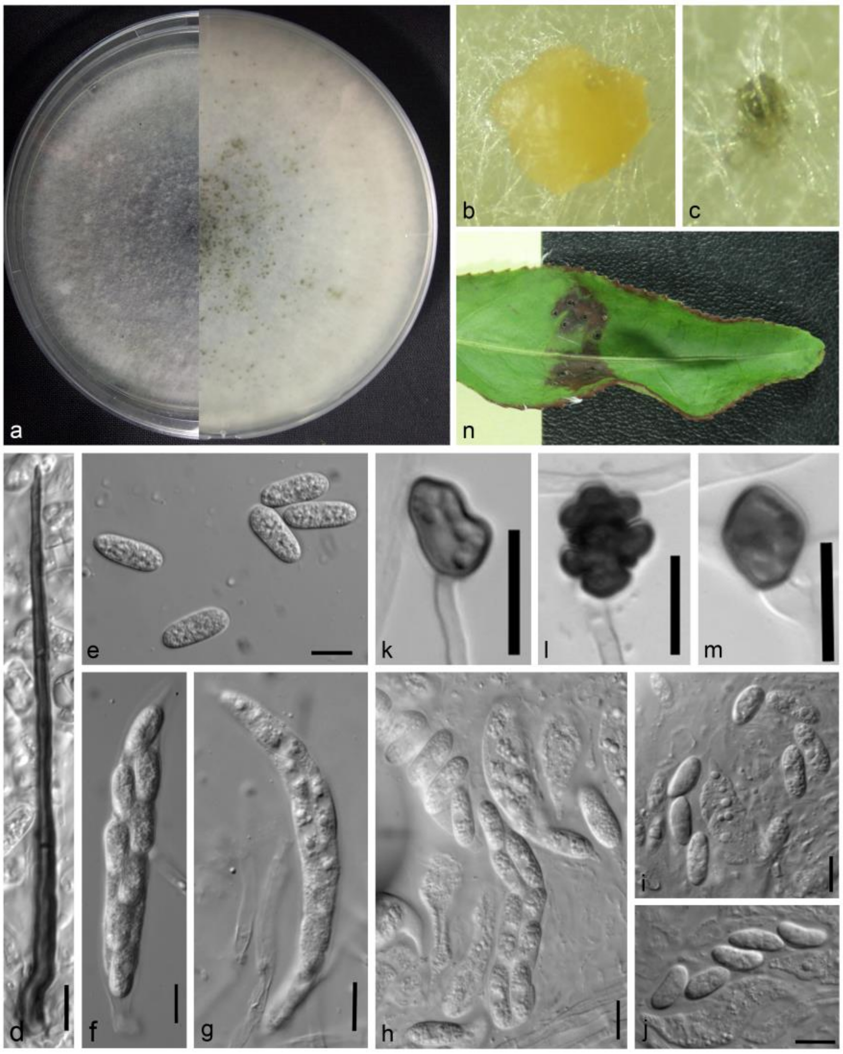

Supplementary Fig. S1 *Colletotrichum aenigma* (from strain JS1A29). a. colony, upper and reverse; b. acervuli; c. ascomata; d. seta; e. conidia; f-h. asci; i-j. ascospores; k-m. appressoria; n. induced symptoms on leaf after 14 days. a-j. from PDA agar medium in 7 days; k-m. from SNA agar medium. Scale: 10 μm

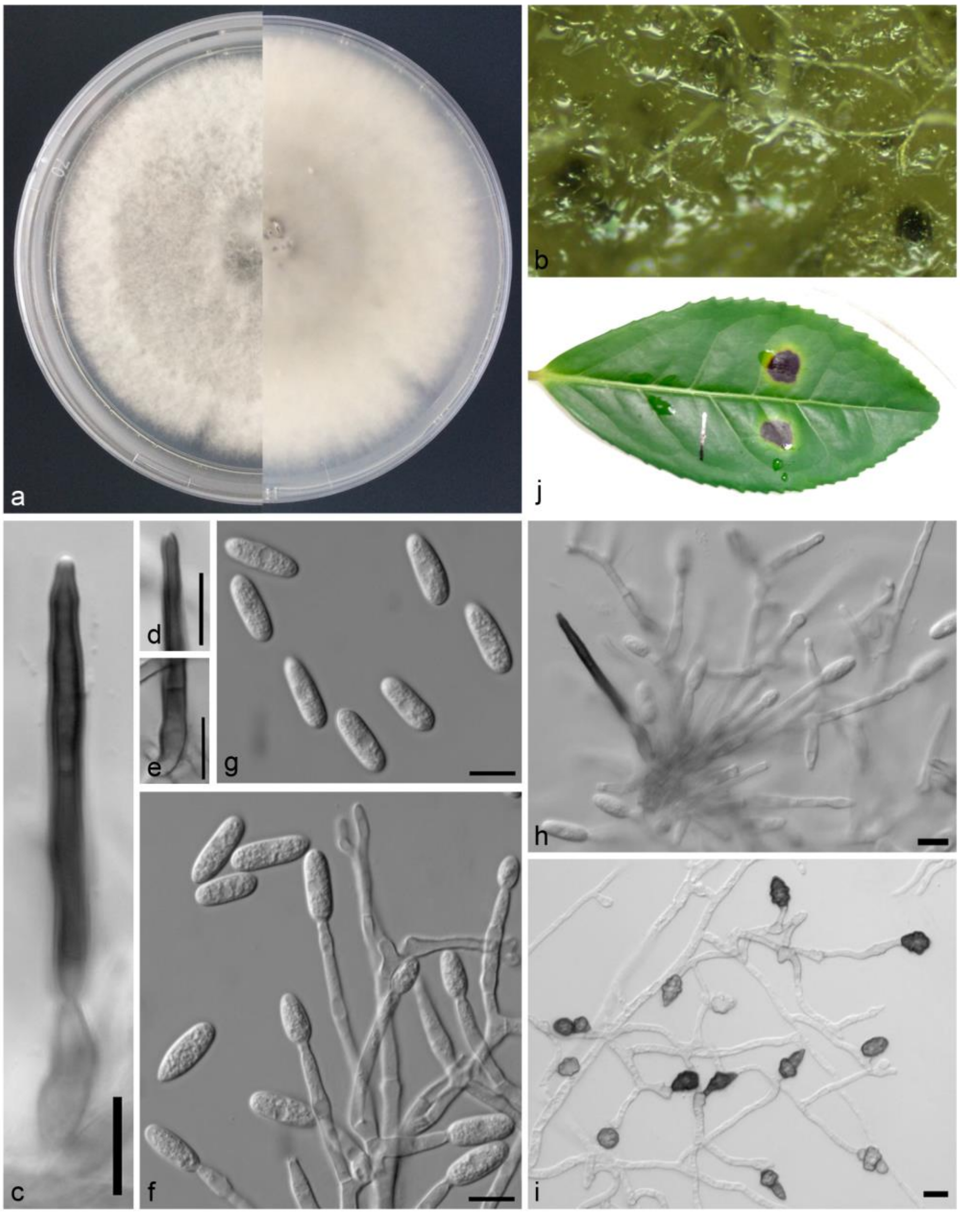

Supplementary Fig. S2 *Colletotrichum camelliae* (from strain JS1A35). a. colony, upper and reverse; b. Chlamydospores; c-e. seta; f. conidiophores; g. conidia; h. conidiophores and seta; i. appressoria; j. induced symptoms on leaf after 14 days. a-h. from PDA agar medium in 7 days; i. from SNA agar medium. Scale: 10 μm

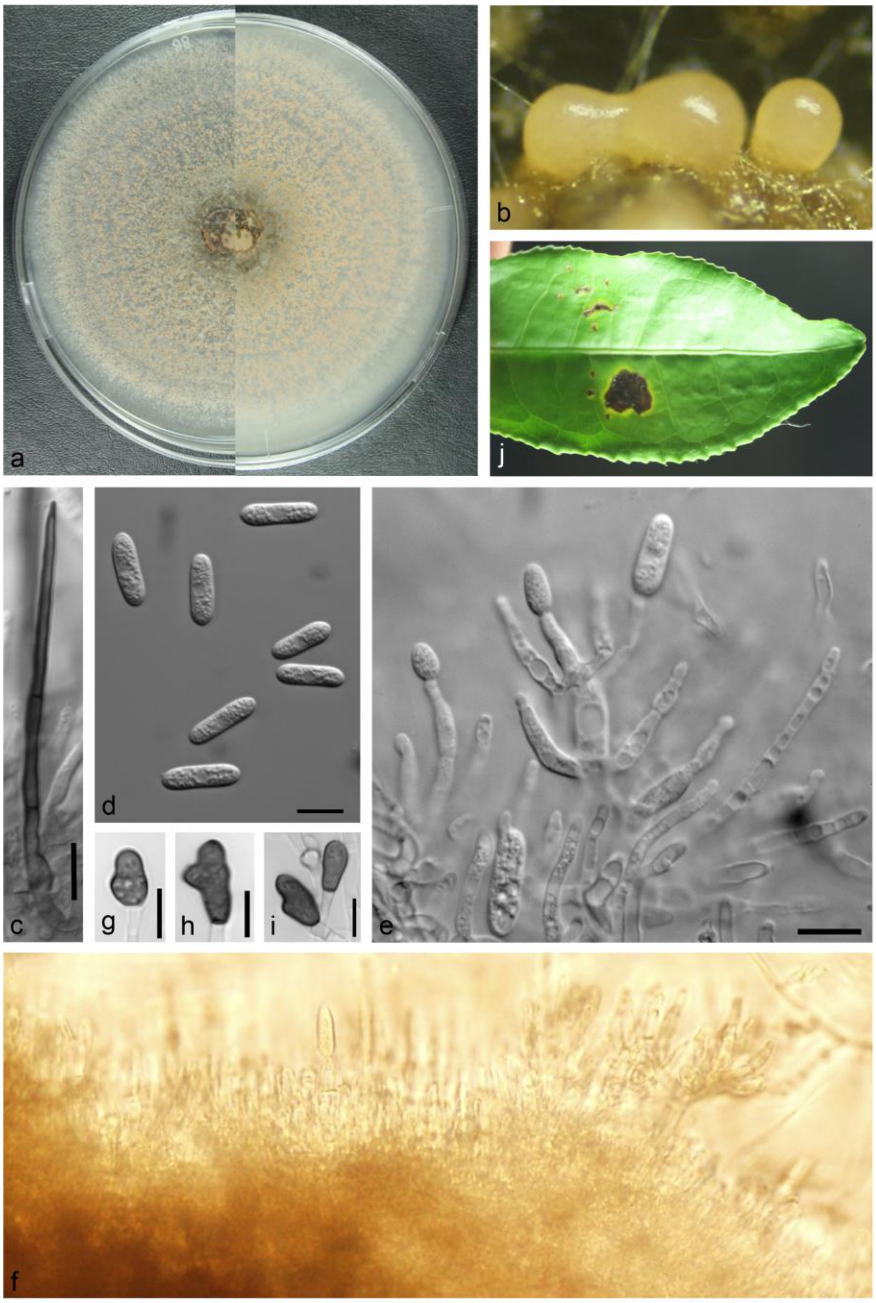

Supplementary Fig. S3 *Colletotrichum endophytica* (from strain YN1A4). a. colony, upper and reverse; b. acervular; c. seta; d. conidia; e-f. conidiophores; g-i. appressoria; j. induced symptoms on leaf after 14 days. a-f. from PDA agar medium in 7 days; g-i. from SNA agar medium. Scale: 10 μm

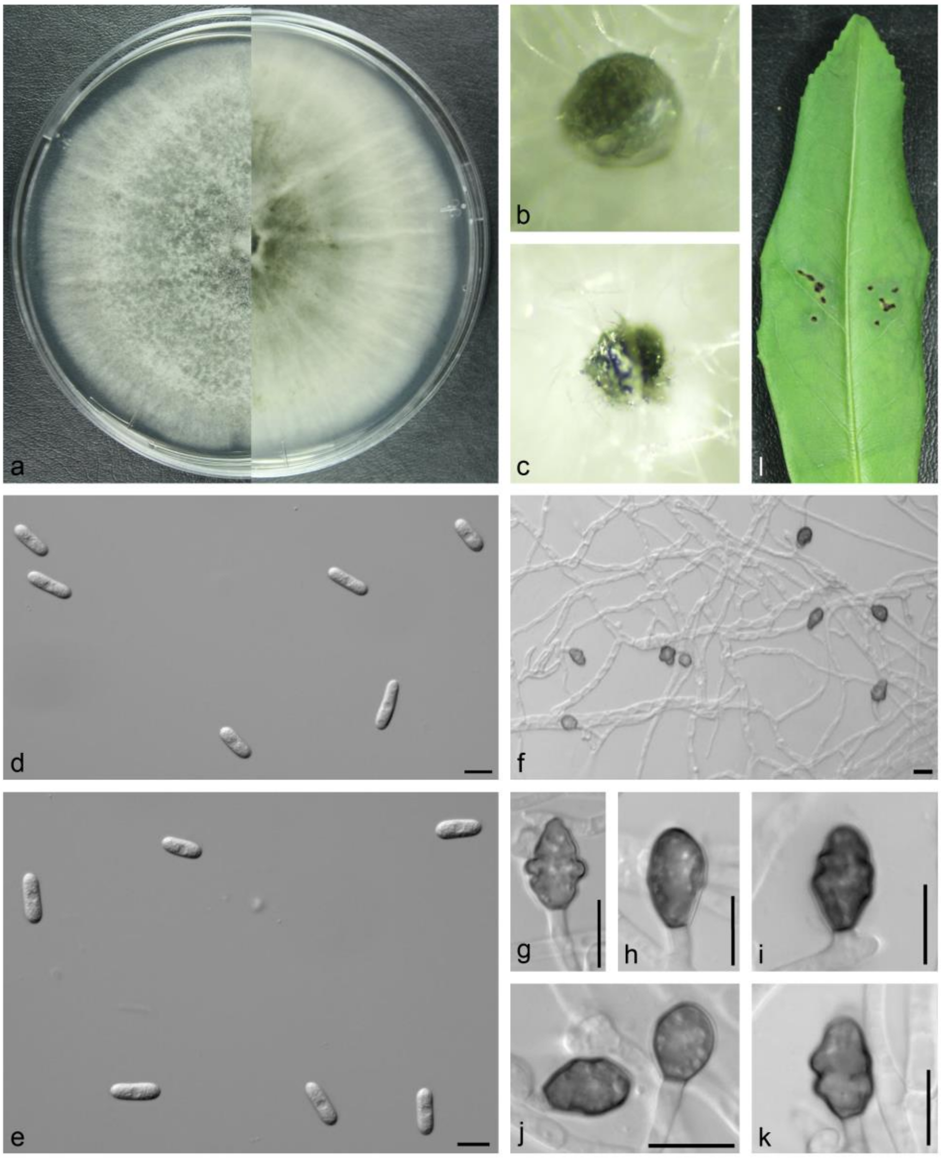

Supplementary Fig. S4 *Colletotrichum* sp. (from strain SC3A3). a. colony, upper and reverse; b-c. Chlamydospores; d-e. conidia; f-k. appressoria; l. induced symptoms on leaf after 14 days. a-e. from PDA agar medium in 7 days; f-k. from SNA agar medium. Scale: 10 μm

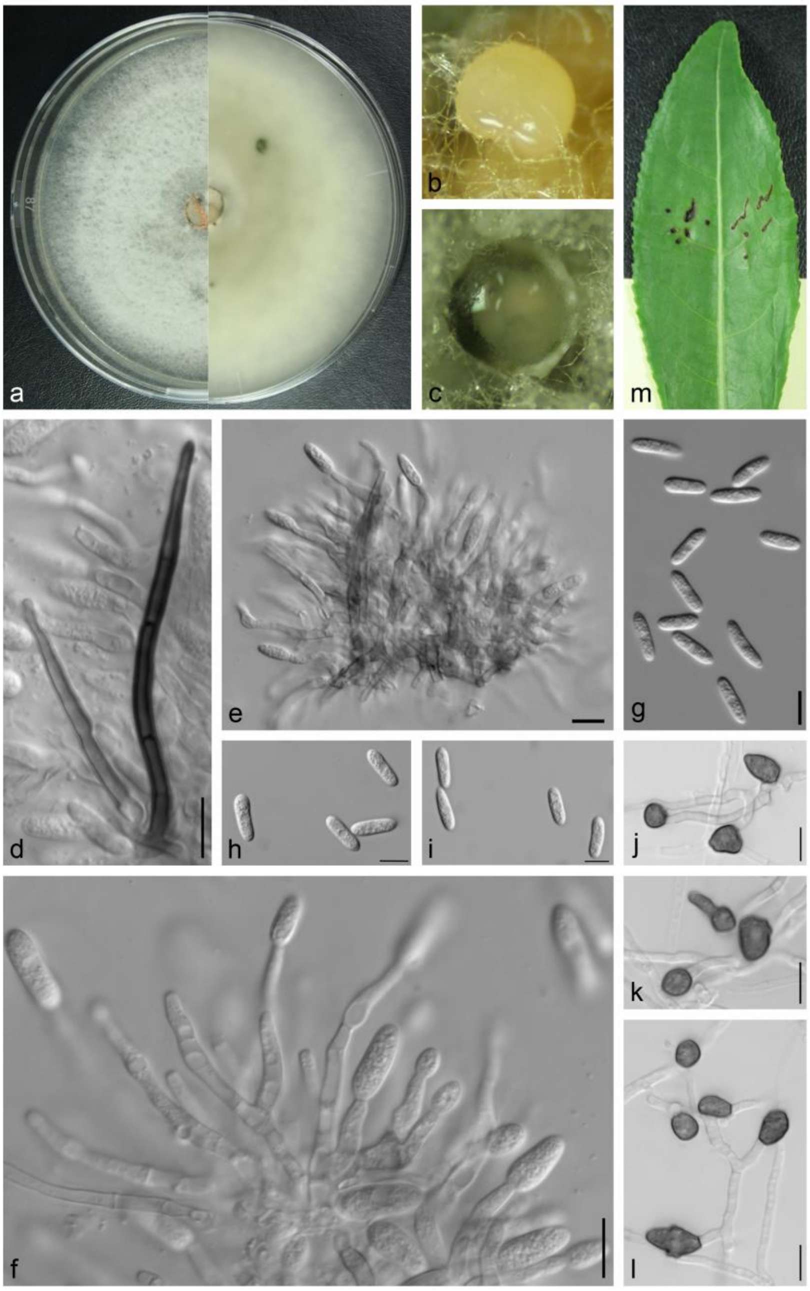

Supplementary **Fig. S5** *Colletotrichum siamense* (from strain GX2A3). a. colony, upper and reverse; b-c. acervular; d. seta; e-f. conidiophores; g-i. conidia; j-l. appressoria; m. induced symptoms on leaf after 14 days. a-i. from PDA agar medium in 7 days; j-l. from SNA agar medium. Scale: 10 μm

Reference

1 Carbone, I. & Kohn, L. M. A method for designing primer sets for speciation studies in filamentous ascomycetes. *Mycologia*, 553-556 (1999).

2 Silva, D. N. *et al.* Application of the Apn2/MAT locus to improve the systematics of the *Colletotrichum gloeosporioides* complex: an example from coffee (*Coffea* spp.) hosts. *Mycologia* **104**, 396-409 (2012).

3 Weir, B. S., Johnston, P. R. & Damm, U. The *Colletotrichum gloeosporioides* species complex. *Stud. Mycol.* **73**, 115-180 (2012).

4 Templeton, M. D., Rikkerink, E. H., Solon, S. L. & Crowhurst, R. N. Cloning and molecular characterization of the glyceraldehyde-3-phosphate dehydrogenase-encoding gene and cDNA from the plant pathogenic fungus *Glomerella cingulata*. *Gene* **122**, 225-230 (1992).

5 Stephenson, S.-A., Green, J. R., Manners, J. M. & Maclean, D. J. Cloning and characterisation of glutamine synthetase from *Colletotrichum gloeosporioides* and demonstration of elevated expression during pathogenesis on *Stylosanthes guianensis*. *Curr. Genet.* **31**, 447-454 (1997).

6 Gardes, M. & Bruns, T. D. ITS primers with enhanced specificity for basidiomycetes-application to the identification of mycorrhizae and rusts. *Mol. Ecol.* **2**, 113-118 (1993).

7 White, T. J., Bruns, T., Lee, S. & Taylor, J. Amplification and direct sequencing of fungal ribosomal RNA genes for phylogenetics. *PCR Protoc.: Guide Methods Appl.* **18**, 315-322 (1990).

8 O'Donnell, K. & Cigelnik, E. Two divergent intragenomic rDNA ITS2 types within a monophyletic lineage of the fungus fusarium are nonorthologous. *Mol. Phylogenet. Evol.* **7**, 103-116 (1997).

9 Glass, N. L. & Donaldson, G. C. Development of primer sets designed for use with the PCR to amplify conserved genes from filamentous ascomycetes. *Appl. Environ. Microbiol.* **61**, 1323-1330 (1995).
